# Neurobiological mechanisms and brain network dysfunction in major depressive disorder: an integrative review

**DOI:** 10.3389/fnint.2026.1904444

**Published:** 2026-09-29

**Authors:** Dina Iskakova, Burcu Yener, Altyngul Kamzanova, Aygul Sitakanova, Rifat Hamoudi, Almira Kustubayeva

**Affiliations:** 1 Department of Biophysics, Biomedicine, and Neuroscience, Al-Farabi Kazakh National University, Almaty, Kazakhstan; 2 Research Institute of Medical and Health Sciences, University of Sharjah, Sharjah, United Arab Emirates; 3 Department of Psychiatry and Narcology, S.D. Asfendiyarov Kazakh National Medical University, Almaty, Kazakhstan; 4 Department of Clinical Science, College of Medicine, University of Sharjah, Sharjah, United Arab Emirates; 5 Biomedically Informed Artificial Intelligence Laboratory (BIMAI-Lab), University of Sharjah, Sharjah, United Arab Emirates; 6 Division of Surgery and Interventional Science, University College London, London, United Kingdom; 7 Brain Institute, Al-Farabi Kazakh National University, Almaty, Kazakhstan

**Keywords:** functional connectivity, genetics, MDD (major depressive disorder), molecular mechanism, neuroimaging

## Abstract

Major depressive disorder (MDD) is a prevalent and disabling psychiatric disorder characterized by persistent mood disturbances, cognitive impairments, and significant systemic consequences. Despite its substantial clinical burden, diagnosis remains challenging because current approaches rely largely on subjective symptom-based assessments and are complicated by marked biological and clinical heterogeneity. In this review, we synthesize findings from neuroimaging, electrophysiology, genetics, and molecular studies to provide an integrated overview of the structural, functional, and neurobiological alterations that underlie MDD. We discuss abnormalities involving large-scale brain networks, neuroplasticity and stress-related pathways, neuroinflammation, oxidative stress, and neurotransmitter systems, together with their implications for biomarker discovery, patient stratification, and treatment response. Collectively, current evidence implicates both molecular dysregulation and large-scale neural network dysfunction in MDD, although direct evidence linking alterations across these biological levels remains limited. Integrating multimodal biological and neuroimaging approaches may improve diagnostic precision, facilitate personalized therapeutic strategies, and advance the development of biologically informed psychiatry.

## 1 Introduction

Major Depressive Disorder (MDD), also known as clinical depression, is a severe and multifactorial psychiatric disorder characterized by persistent emotional distress. The core clinical symptoms include a persistently depressed or irritable mood, a marked decrease in interest or pleasure in previously enjoyed activities (anhedonia), changes in appetite or weight, sleep disturbances (e.g., insomnia or hypersomnia), fatigue, feelings of worthlessness or excessive guilt, difficulty concentrating, and recurrent thoughts of death or suicide (American Psychiatric Association, 2013). In addition to these diagnostic features, MDD is associated with broader systemic effects involving the cardiovascular, immune, gastrointestinal, endocrine, and neurological systems (Chen D. et al., 2023; Ismail et al., 2017; Jiao et al., 2025; Vaccarino et al., 2020). The clinical features and multisystem impact of MDD are summarized in Figure 1.

**Figure 1:**
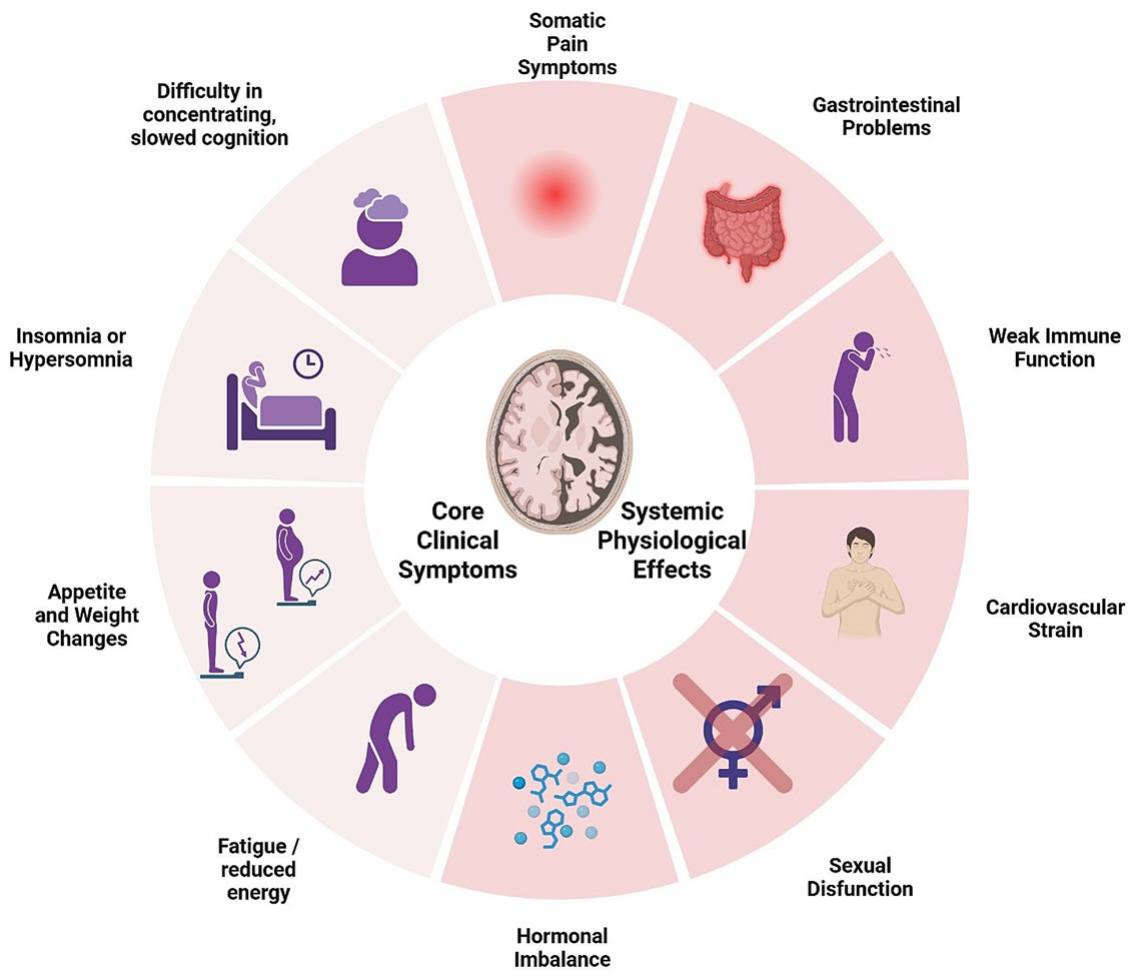
Clinical features and multisystem impact of major depressive disorder (MDD). The figure distinguishes core clinical symptoms used for diagnosis (e.g., mood, sleep, appetite, cognition) from broader systemic physiological effects associated with MDD, including immune, hormonal, and cardiovascular alterations. (Created in https://BioRender.com).

The development of MDD is influenced by a wide range of interacting biological, psychological, and environmental risk factors. These include genetic susceptibility, dysregulation of neurobiological systems such as the hypothalamic–pituitary–adrenal (HPA) axis, chronic inflammation, oxidative stress, early-life adversity, personality traits, and stressful life events (Kendler et al., 2002; Otte et al., 2016). The multifactorial etiology of MDD reflects the complex interactions among these risk factors across molecular, cellular, and neural network levels, contributing to substantial clinical and biological heterogeneity. The main risk factors associated with MDD are summarized in Figure 2. The Diagnostic and Statistical Manual of Mental Disorders, Fifth Edition (DSM-5) defines MDD based on the presence of specific clinical symptoms persisting for at least 2 weeks and causing significant impairment in daily functioning (American Psychiatric Association, 2013). However, these symptoms may overlap with normal emotional fluctuations or developmental changes, particularly in adolescents and young adults, complicating accurate diagnosis. In addition, clinical assessment may be hindered by underreporting of symptoms due to social stigma, limited mental health awareness, or difficulties in articulating internal emotional states.

**Figure 2:**
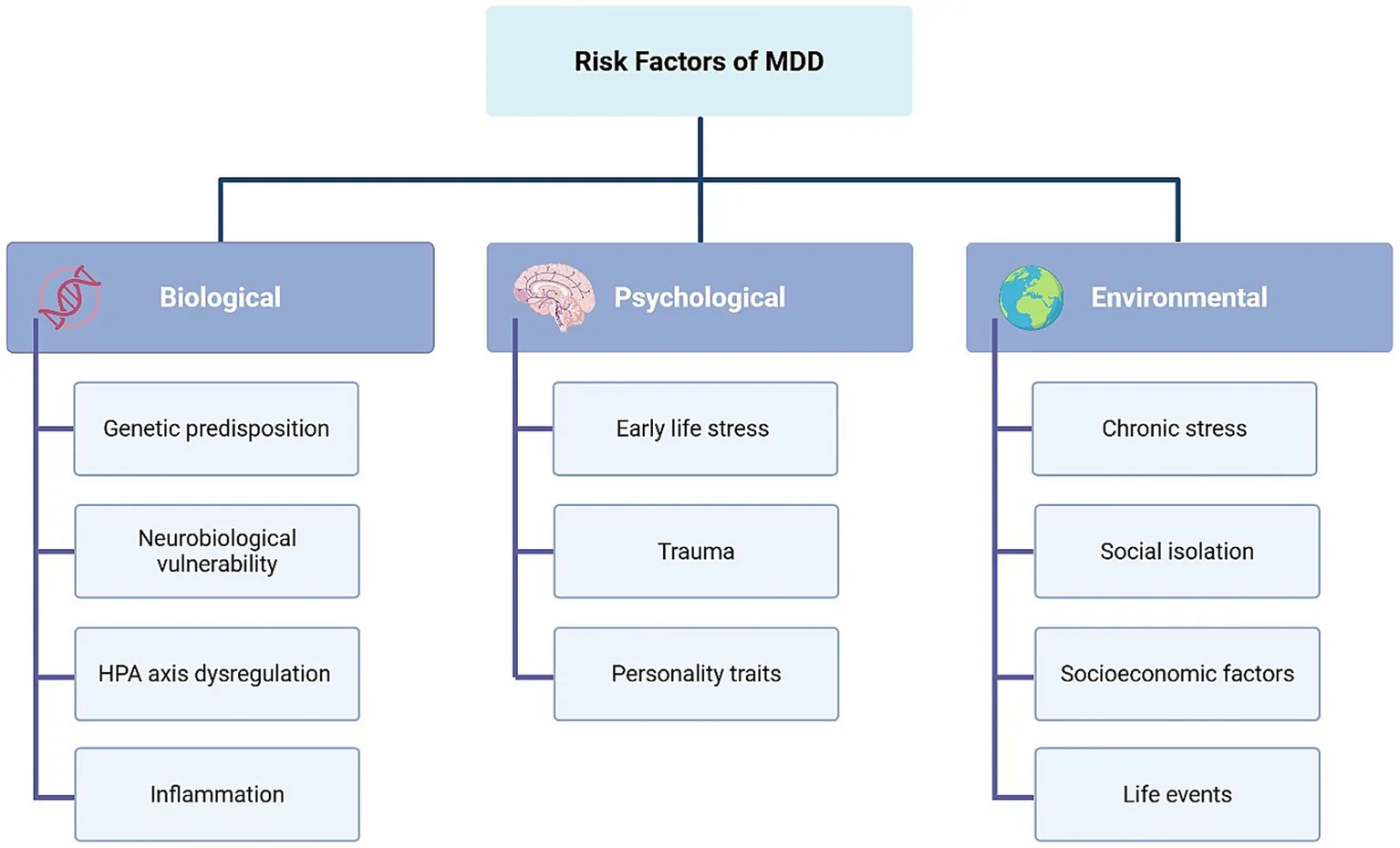
Risk factors contributing to the development of MDD. Risk factors are categorized into biological, psychological, and environmental domains, reflecting the multifactorial etiology of MDD.

According to the Global Burden of Disease (GBD 2019 Mental Disorders Collaborators, 2022) study, depression affects approximately 280 million people worldwide. Despite its high prevalence, current diagnostic approaches for MDD are mainly based on subjective assessments, including DSM-5-based diagnostic criteria, self-report questionnaires, and clinical interviews. In clinical practice, although diagnosis is typically performed by psychiatrists, limited access to specialized mental health care, means that many patients are assessed by general practitioners. Commonly used screening tools and questionnaires are susceptible to reporting bias and may inadequately capture the clinical heterogeneity observed across different patient subgroups and disease stages (Nelson et al., 2018). Additionally, these tools may be insufficient for distinguishing MDD from comorbid psychiatric conditions or for evaluating treatment status. Collectively, these limitations highlight the urgent need for objective biological markers to improve diagnostic precision and support personalized treatment strategies in MDD (Fried and Nesse, 2015; Insel and Cuthbert, 2015). Promising approaches, such as electroencephalography (EEG), neuroimaging, genetic profiling, and peripheral biomarker-based analyses have demonstrated potential for identifying biologically relevant alterations associated with MDD. However, when used individually, these methods often show limited reproducibility, insufficient specificity, and restricted clinical applicability for routine psychiatric practice (Woo et al., 2017).

In this context, the present review provides a comprehensive overview of current neurobiological and diagnostic approaches in MDD, with a particular focus on neuroimaging, molecular biology, genomics, electrophysiology, and biomarker-based analyses. Particular attention is given to emerging objective and data-driven strategies that may contribute to biologically informed diagnosis and personalized treatment approaches in MDD.

## 2 Literature search strategy

A comprehensive literature search was conducted using PubMed, Scopus, and the Web of Science Core Collection, covering the period from database inception to March 2026. Google Scholar was additionally consulted for citation tracking and full-text retrieval. An updated search was conducted on 28 July 2026 to identify recent studies. The search focused on peer-reviewed literature examining molecular, genetic, neuroendocrine, neuroimmune, neurotransmitter, neuroplastic, and structural-functional brain alterations associated with major depressive disorder (MDD).

Search terms included “major depressive disorder,” “MDD,” and “depression” which were combined using Boolean operators (AND/OR) with terms related to the principal mechanisms considered in this review, including “BDNF,” “neuroplasticity,” “HPA axis,” “cortisol,” “monoamines,” “serotonin,” “dopamine,” “norepinephrine,” “GABA,” “glutamate,” “neuroinflammation,” “cytokines,” “microglia,” “NF-κB,” “NLRP3,” “JAK/STAT,” “oxidative stress,” “Nrf2,” “genetics,” “polymorphism,” and “epigenetics.” These terms were combined, where relevant, with neuroimaging and electrophysiological terms including “MRI,” “fMRI,” “PET,” “MRS,” “DTI,” “EEG,” “functional connectivity,” “structural connectivity,” and “brain networks.” Search terms were adapted to each database, and targeted searches were conducted for individual mechanisms. Reference lists of relevant original studies, systematic reviews, and meta-analyses were also screened for additional publications.

Peer-reviewed original studies, systematic reviews, and meta-analyses published in English were considered when they addressed MDD or biological mechanisms relevant to its neurobiology. Human studies were prioritized for clinical, molecular, genetic, and neuroimaging evidence, while preclinical studies were selectively included when they provided mechanistic information not readily obtainable in humans. Seminal mechanistic and methodological studies not restricted to MDD were also included to describe established biological pathways or theoretical frameworks relevant to MDD. Conference abstracts, editorials, letters, dissertations, non-peer-reviewed publications, duplicate reports, and studies primarily addressing other psychiatric or neurological disorders without findings relevant to MDD were excluded.

Study selection involved initial screening of titles and abstracts followed by full-text assessment of relevant publications. Evidence from human neuroimaging, peripheral biomarkers, genetic and genomic investigations, postmortem studies, and preclinical models was distinguished where appropriate. Conflicting and null findings were retained to reflect heterogeneity in the evidence. As this was an integrative narrative review rather than a systematic review or meta-analysis, no quantitative meta-analysis or formal PRISMA-based study-selection procedure was undertaken.

## 3 Integration of neuroimaging and molecular findings

MDD is increasingly recognized as a multifactorial disorder arising from complex interactions between molecular, cellular, and large-scale neural network-level abnormalities. Consequently, multimodal approaches integrating neuroimaging, molecular biomarkers, electrophysiology, and genetic data have gained increasing attention because of their potential to improve biological characterization of MDD, facilitate patient stratification, and support the development of more personalized therapeutic strategies. Multiple biological hypotheses have been proposed to explain the development and progression of MDD, including neurotrophic dysregulation, monoaminergic imbalance, oxidative stress, inflammation, and chronic stress-related mechanisms. Although each of these hypotheses provides valuable insights into specific aspects of the disorder, none independently explains the substantial heterogeneity and clinical variability observed among patients with MDD. Importantly, evidence supporting these mechanisms varies considerably in terms of study population, biological material, experimental approach, and strength of association with clinical phenotypes. To provide a structured framework for interpreting the evidence across molecular and neuroimaging levels, Table 1 presents an evidence matrix summarizing molecular or genetic alterations, available multimodal evidence directly linking molecular and neuroimaging findings, and areas in which such evidence remains lacking. Importantly, the matrix distinguishes direct multimodal evidence obtained within the same studies from converging molecular and neuroimaging findings derived from separate study populations, thereby avoiding inference of direct molecular–brain relationships where these have not been empirically demonstrated. Neuroimaging techniques provide complementary tools for investigating the structural and functional brain alterations associated with MDD. Structural magnetic resonance imaging (MRI) has revealed abnormalities in gray matter volume, cortical thickness, and white matter integrity, particularly within prefrontal-limbic circuits (Drevets et al., 2008; Schmaal et al., 2016). More broadly, structural alterations have been reported in the hippocampus, prefrontal cortex, anterior cingulate cortex, amygdala, thalamus, and other cortical and subcortical regions, although the magnitude and regional distribution of these findings vary across studies and clinical populations (Arnone et al., 2013; Grieve et al., 2013; Zhang F. et al., 2018).

**Table 1:** Evidence matrix integrating molecular and genetic findings with structural and functional neuroimaging findings in MDD.

Biological domain	Pathway/Molecular or genetic marker	Molecular/Genetic finding	Molecular-neuroimaging evidence	Evidence gap/Interpretation
Neurotrophic signaling	BDNF/TrkB–PI3K/Akt signaling; BDNF Val66Met; BDNF promoter methylation	Altered BDNF signaling, including reduced peripheral BDNF levels and functional effects of the BDNF Val66Met polymorphism on activity-dependent BDNF secretion (Molendijk et al., 2014)	Serum BDNF–cortical thickness/gray matter volume (Polyakova et al., 2020) BDNF promoter methylation–cortical thickness (Na et al., 2016) Serum BDNF–resting-state functional connectivity (Chen S. et al., 2023) BDNF Val66Met–structural covariance and resting-state functional connectivity (Wheeler et al., 2018)	Direct multimodal evidence remains limited and heterogeneous; peripheral BDNF measures should not be considered direct surrogates of central BDNF signaling, and the available associations do not establish causal molecular-to-brain relationships. Direct multimodal evidence specifically linking proBDNF–p75NTR signaling to neuroimaging findings is currently lacking.
Neurotrophic signaling	MAPK/ERK, PI3K/Akt, ARC	Reduced ARC expression has been reported in transcriptomic and postmortem studies of MDD (Gammie, 2021; Yu et al., 2022), while alterations involving PI3K/Akt and MAPK signaling have also been reported in association with MDD-related phenotypes (Fujii et al., 2011; Gao et al., 2021; Losenkov et al., 2016; Yang, 2012).	No studies identified that simultaneously assessed ARC expression and neuroimaging measures in the same participants.	Direct molecular–neuroimaging evidence is currently lacking; reported effects on synaptic plasticity, dendritic spine maintenance, and long-term potentiation are based on cellular/functional rather than neuroimaging evidence.
Stress / neuroendocrine signaling	HPA axis: CRH, ACTH, cortisol, glucocorticoid receptor (NR3C1)	HPA-axis dysregulation, including elevated CRH, ACTH and cortisol and impaired glucocorticoid receptor-mediated feedback (Ceruso et al., 2020; Kageyama et al., 2021; Shim et al., 2022; Van West et al., 2006).	Serum cortisol associated with cortical thickness (Liu et al., 2015), White-matter integrity (Liu et al., 2016) Hippocampal morphology (Watanabe et al., 2017) Structural brain-network alterations (Nguyen et al., 2020) HPA-axis normalization ↔ changes in prefrontal and limbic/paralimbic neural activity (Aihara et al., 2007)	Available multimodal evidence supports cortisol–brain structural associations, although HPA-axis abnormalities remain heterogeneous across MDD populations.
Neuroinflammation	NF-κB / NLRP3 inflammasome	Activation of NF-κB and NLRP3 inflammasome signaling, with increased pro-inflammatory cytokine signaling and enhanced maturation of IL-1β and IL-18 (Guo et al., 2015; Liu et al., 2017; Taene et al., 2020).	No direct multimodal studies identified that assessed NF-κB/NLRP3 signaling together with neuroimaging measures in MDD.	Most mechanistic evidence derives from experimental models or peripheral biomarkers; direct *in vivo* human molecular–neuroimaging evidence is lacking, and the magnitude of NLRP3 activation varies across studies.
Neuroinflammation	JAK/STAT signaling	Dysregulated JAK/STAT signaling has been implicated in MDD and linked to inflammatory signaling and antidepressant-related molecular responses (Al-Samhari et al., 2016; Gałecka et al., 2022; Shariq et al., 2018).	No direct multimodal studies identified that specifically assessed JAK/STAT signaling together with neuroimaging measures in MDD.	Evidence is derived predominantly from molecular and experimental studies; direct in vivo human molecular–neuroimaging evidence remains lacking, and findings across JAK/STAT-related studies are heterogeneous.
Neuroinflammation	Peripheral cytokines (IL-1β, IL-6, IL-18, TNF-α)	Elevated pro-inflammatory cytokines, including IL-1β, IL-6, IL-18, and TNF-α, have been reported in MDD, although elevations are present only in a subset of patients (Köhler et al., 2017; Osimo et al., 2020, 2019; Poletti et al., 2024).	Blood inflammatory markers ↔ functional network dysconnectivity (Aruldass et al., 2021). Peripheral inflammation ↔ altered structural and functional brain-network connectivity (Kitzbichler et al., 2021).	Inflammatory profiles are heterogeneous across MDD populations; peripheral cytokine abnormalities should not be considered a universal feature of MDD.
Monoaminergic/metabolic signaling	Tryptophan–kynurenine pathway/serotonin	Altered tryptophan metabolism toward the kynurenine pathway, with reduced serotonin synthesis and altered balance between neuroactive kynurenine metabolites, including quinolinic and kynurenic acids (Correia and Vale, 2022; Gong et al., 2023).	Serotonin transporter (SERT) availability ↔ MRI/fMRI measures (James et al., 2017; Klöbl et al., 2025) No direct multimodal evidence identified linking kynurenine-pathway metabolites with the reported structural or functional brain alterations in MDD.	Direct evidence linking kynurenine-pathway alterations to neuroimaging abnormalities remains lacking; serotonergic molecular-neuroimaging findings are also heterogeneous across studies.
Monoaminergic signaling	Dopamine; dopamine receptors/transporters (e.g., SLC6A3)	Altered dopaminergic signaling and genetic variation in dopamine receptors/transporters, including SLC6A3, have been associated with MDD; altered PRKCB methylation has also been reported in first-episode, drug-naïve MDD (Reith et al., 2022; Tao et al., 2024).	Reduced striatal dopamine release ↔ decreased striatal functional connectivity / impaired reward-network function (Hamilton et al., 2018) Reduced dopamine receptor availability ↔ decreased striatal functional connectivity / impaired reward-network function (Schneier et al., 2018)	Available multimodal studies assess central dopaminergic function using molecular neuroimaging; relationships between peripheral dopamine levels and structural or functional brain abnormalities remain poorly characterized
Inhibitory neurotransmission	GABA/GABAergic signaling	Reduced expression of GABA-related genes has been reported in postmortem transcriptomic studies of MDD (Oh et al., 2022).	Reduced GABA concentrations in the PFC and ACC measured by ^1^H-MRS (Godfrey et al., 2018; Ritter et al., 2022). No direct studies identified linking peripheral or transcriptomic GABAergic markers with structural or functional MRI findings.	^1^H-MRS provides in vivo evidence of altered central GABA levels, but direct integration of molecular/peripheral GABAergic markers with structural or functional neuroimaging remains limited; reported GABA abnormalities also vary by illness stage, brain region, and methodology.
Excitatory neurotransmission	Glutamate/NMDA–AMPA signaling	Dysregulated NMDA/AMPA receptor signaling and altered glutamatergic neurotransmission, with heterogeneous glutamate/glutamine abnormalities in MDD (Duman and Aghajanian, 2012; Godlewska et al., 2018; Krystal et al., 2013).	Serum glutaminase ↔ ACC glutamate measured by ^1^H-MRS (Wise et al., 2018) Glutamatergic PET measures ↔ MRS neurochemical measures (Abdallah et al., 2017)	Glutamatergic findings are heterogeneous across studies and may vary according to brain region, illness stage, and imaging methodology.
Oxidative stress	Nrf2/Keap1/ARE	Altered Nrf2/Keap1 signaling and impaired antioxidant defense, supported predominantly by preclinical evidence (Hashimoto, 2018; Yao et al., 2016).	No direct multimodal studies identified specifically assessing Nrf2/Keap1/ARE signaling together with neuroimaging outcomes in MDD.	Evidence is predominantly preclinical; direct in vivo human Nrf2/Keap1–neuroimaging evidence is lacking.

ACTH, adrenocorticotropic hormone; AKT, protein kinase B; ARC, activity-regulated cytoskeleton-associated protein; ARE, antioxidant response element; BDNF, brain-derived neurotrophic factor; CRH, corticotropin-releasing hormone; DMN, default mode network; ERK, extracellular signal-regulated kinase; fMRI, functional magnetic resonance imaging; GABA, gamma-aminobutyric acid; GMV, gray matter volume; ^1^H-MRS, proton magnetic resonance spectroscopy; HPA, hypothalamic–pituitary–adrenal axis; IL, interleukin; JAK/STAT, Janus kinase/signal transducer and activator of transcription; Keap1, Kelch-like ECH-associated protein 1; MAPK, mitogen-activated protein kinase; MDD, major depressive disorder; MRI, magnetic resonance imaging; mTOR, mechanistic target of rapamycin; NF-κB, nuclear factor kappa B; NLRP3, NOD-like receptor family pyrin domain-containing 3; NMDA, N-methyl-D-aspartate; NR3C1, glucocorticoid receptor; PET, positron emission tomography; PI3K, phosphoinositide 3-kinase; proBDNF, precursor brain-derived neurotrophic factor; p75NTR, p75 neurotrophin receptor; ROS, reactive oxygen species; TNF-α, tumor necrosis factor alpha; TrkB, tropomyosin receptor kinase B.

Functional MRI studies have reported abnormalities of the default mode network (DMN), salience network (SN), and central executive network (CEN), which are considered core large-scale brain networks disrupted in MDD (Bullmore and Sporns, 2009; Kaiser et al., 2015; Menon, 2011). Functional neuroimaging studies have also reported altered regional activity and functional connectivity within prefrontal–limbic and reward-related circuits involved in emotional processing, cognitive control, and reward processing (Fitzgerald et al., 2008; Hamilton et al., 2012; Scheepens et al., 2020). These findings indicate that MDD is associated with alterations extending beyond individual brain regions to distributed neural systems; however, the direction and magnitude of these abnormalities differ according to clinical characteristics, treatment exposure, imaging methodology, and analytical approach.

Electroencephalography (EEG), with its high temporal resolution, provides information about the temporal organization of neural activity. EEG studies in MDD have reported alterations in oscillatory activity, functional connectivity, neural synchronization, and microstate dynamics (Olbrich and Arns, 2013; Xue et al., 2024; Yuan et al., 2024). Positron emission tomography (PET) has been used to evaluate neurotransmitter systems, neuroinflammation, and metabolic activity (Meyer, 2012). Proton magnetic resonance spectroscopy (^1^H-MRS) provides an additional approach for assessing regional neurochemical metabolites, including glutamate, glutamine, and gamma-aminobutyric acid. PET and ^1^H-MRS therefore permit a more direct assessment of selected molecular and neurochemical processes than conventional structural MRI or fMRI, although their findings remain dependent on the specificity of the tracer, metabolite, brain region, and analytical methodology employed. These modalities therefore characterize complementary aspects of MDD neurobiology, but neuroimaging abnormalities alone cannot establish the molecular mechanisms underlying the observed structural or functional changes.

Taken together, molecular and neuroimaging studies provide converging evidence suggesting that abnormalities at the molecular, cellular, and systems levels are associated with the pathophysiology of MDD. However, much of this evidence originates from separate study populations, and relatively few studies have directly assessed molecular or genetic measures together with neuroimaging outcomes in the same participants. Consequently, apparent correspondence between molecular and neuroimaging findings should generally be interpreted as converging evidence rather than evidence of a direct molecular-to-brain relationship. Furthermore, heterogeneity in clinical subtype, illness stage and severity, treatment exposure, biological specimens, molecular assays, and neuroimaging methodologies may contribute to differences across studies. An important unresolved question is therefore whether the reported molecular and brain abnormalities represent interconnected components of common biological mechanisms, characterize distinct biological subgroups of MDD, or partly reflect differences in study populations and methodologies.

The following sections critically examine the major biological mechanisms implicated in MDD, with particular attention to the consistency and heterogeneity of findings, the level of evidence supporting molecular–neuroimaging relationships, and current gaps in the literature. Table 2 provides a study-level summary of representative neuroimaging and multimodal studies, including relevant sample and clinical characteristics, imaging findings, and reported clinical associations. A more comprehensive study-level summary of neuroimaging and multimodal evidence is provided in Supplementary Table S1.

**Table 2:** Representative neuroimaging and multimodal studies in major depressive disorder: study characteristics, imaging findings, and clinical associations.

Study	Sample/clinical Characteristics	Imaging modality	Brain region/Network	Main imaging finding	Clinical association reported
A. Primary clinical, treatment, familial-risk and multimodal cohort studies					
Abdallah et al. (2017)	30 medication-free MDD; 35 HC (PET). Subset: 16 MDD; 23 HC (^1^H-MRS).	[^18^F]FPEB PET + ^1^H-MRS	ACC; whole-brain mGluR5	No group difference in mGluR5; MDD had higher ACC glutamate, glutamine and Glx. mGluR5 availability inversely related to glutamate/Glx.	Direct-PET–MRS association tested in the same subset; no symptom-severity association reported.
Aihara et al. (2007)	24 antidepressant-free MDD; 10 treatment responders rescanned; HC comparison (n NR in abstract).	[^18^F]FDG-PET + DEX/CRH test	Medial PFC; hippocampal/parahippocampal regions	Frontal hypometabolism and right hippocampal/parahippocampal hypermetabolism; abnormalities normalized in responders.	Direct-metabolism related to HPA-axis status and treatment response in this cohort.
Arnone et al. (2013)	39 medication-free current unipolar depression; 25 medication-free remitted; 66 HC. Thirty-two current cases received citalopram for 8 weeks.	T1 MRI; VBM	Bilateral hippocampus	Lower hippocampal gray matter in current depression; volume increased after treatment/remission.	Direct-state and longitudinal treatment/remission association.
Aruldass et al. (2021)	83 depression (CRP ≤ 3 mg/L, n = 50; >3 mg/L, n = 33); 46 HC.	Resting-state fMRI + blood inflammatory markers	Interoceptive network (insula-centered)	High-CRP depression showed attenuated functional connectivity.	Direct-connectivity associated with CRP/immune markers in this sample.
Grieve et al. (2013)	102 non-geriatric MDD outpatients; 34 matched HC; 31% melancholic; mean illness duration 11.3 years.	T1 MRI; whole-brain VBM and cortical thickness	PFC/ACC; precuneus; temporal, parietal and occipital cortex; medial OFC	Widespread lower gray-matter volume and focal left medial OFC thinning.	Negative-structural alterations were not significantly associated with depressive symptom severity.
Hamilton et al. (2018)	16 depressed participants; 14 HC.	Concurrent [^11^C]raclopride PET + resting-state fMRI	Ventral/dorsal striatum; DMN and salience targets	Higher striatal binding potential in depression; binding covaried with corticostriatal connectivity.	Direct PET–fMRI association; no symptom-severity association reported.
Hannestad et al. (2013)	10 acute mild–moderate MDD; 10 genotype-matched HC.	[^11^C]PBR28 TSPO PET	Whole brain; predefined cortical/subcortical ROIs	No evidence of elevated TSPO; most MDD participants showed lower binding than matched controls.	Negative-no support for increased neuroinflammation; no clinical correlation reported.
Klöbl et al. (2025)	153 participants, including 44 MDD; randomized acute IV citalopram/placebo.	Emotional-face fMRI + [^11^C]DASB PET	ACC; striatum/thalamus; parietal/superior frontal cortex	Citalopram reduced fear-related ACC activation; MDD showed altered frontoparietal activation.	Direct-ACC activation inversely related to SERT availability and drug-related activation changes related to emotional attribution.
Liu et al. (2015)	30 drug-naïve first-episode MDD; 41 matched HC.	Structural MRI; cortical thickness + serum cortisol	Left lateral OFC	MDD showed OFC thinning and higher morning cortisol.	Direct-OFC thickness inversely correlated with cortisol in MDD.
Liu et al. (2024)	57 first-episode drug-naïve MDD; 60 HC; MDD stratified by suicidal ideation.	Resting-state fMRI + plasma oxidative-stress markers	Thalamus, ACC, superior frontal gyrus, insula and precuneus	SOD/GSR related to regional activity and thalamic connectivity; patterns differed by suicidal-ideation status.	Direct biomarker–imaging association; negative for HAMD association.
Na et al. (2016)	65 recurrent MDD; 65 matched HC.	Structural MRI + BDNF promoter methylation	Frontal, temporal, occipital, parietal and sensorimotor cortex	Higher BDNF methylation related to lower cortical thickness across multiple regions in MDD.	Direct epigenetic–imaging association; no treatment-outcome association.
Polyakova et al. (2020)	20 minor depressive episode; 40 HC; first and recurrent episodes represented.	Structural MRI + serum BDNF	OFC, rostral ACC, insula, cingulate and superior frontal cortex	BDNF positively related to regional thickness/volume in depression, driven mainly by first episode.	Direct-but the clinical group had minor depressive episodes, not necessarily DSM MDD.
Schindler et al. (2019)	20 unmedicated MDD; 20 medicated MDD; 21 bipolar disorder; 23 HC.	7-T structural MRI	Hypothalamic subunits	Approximately 5% larger left hypothalamus across patient groups.	Direct diagnosis/medication comparison-effect was independent of medication; no symptom association reported.
Setiawan et al. (2015)	20 medication-free MDD in a major depressive episode; 20 HC.	[^18^F]FEPPA TSPO PET	ACC; PFC; insula	Higher TSPO distribution volume in MDD.	Direct-ACC TSPO was positively associated with depression severity.
Wise et al. (2018)	29 medication-free depression (20 unipolar, 9 bipolar); 20 HC.	^1^H-MRS + peripheral glutaminase assay	Dorsal ACC	Lower ACC glutamate, significant in unipolar depression.	Negative-peripheral glutaminase was not significantly related to central glutamate; clinical sample mixed unipolar/bipolar.
B. Meta-analyses, systematic reviews and imaging-focused narrative reviews					
Kaiser et al. (2015)	Meta-analysis: 27 datasets/25 publications; 556 MDD; 518 HC.	Resting-state fMRI connectivity	DMN, frontoparietal-control and affective networks	Pooled DMN hyperconnectivity and imbalanced control-network connectivity.	Pooled-clinical interpretations are network-level, not participant-level correlations.
Kambeitz and Howes (2015)	Meta-analysis: 50 imaging/post-mortem studies; 877 depression; 968 HC.	PET/SPECT SERT imaging (plus post-mortem)	Striatum, amygdala, brainstem, thalamus and hippocampus	Lower SERT availability in striatum, amygdala and brainstem; no thalamic/hippocampal effect.	Pooled-not a study-specific clinical association.
Pereira et al. (2018)	Systematic review: 65 studies (4,034 MDD; 3,293 HC); meta-analytic subset 22 studies (882 MDD; 616 HC).	Imaging genetics; structural/functional MRI	Hippocampus and distributed gray/white-matter and activation phenotypes	No robust BDNF/5-HTTLPR hippocampal-volume effect; exploratory effects elsewhere.	Pooled-no participant-level clinical association; many findings exploratory.
Schmaal et al. (2016)	Individual-participant meta-analysis: 1,728 MDD; 7,199 HC across 15 cohorts.	Structural MRI; subcortical segmentation	Hippocampus and other subcortical regions	Smaller hippocampus in MDD, driven by recurrent illness; no first-episode effect.	Pooled moderators-early onset/recurrent status related to volume; no severity association.
Schür et al. (2016)	Meta-analysis: 40 studies; 1,591 participants across disorders; MDD n = 437.	^1^H-MRS	Cortical GABA/glutamate voxels	Lower GABA in currently depressed MDD but not remitted MDD.	Pooled state association-not an individual-level symptom correlation.

Evidence labels: Direct = the association was tested in the same participant sample; Negative = the stated clinical association was tested but not supported; Group only = an imaging difference was reported without a study-specific clinical correlation; Pooled = meta-analytic/systematic-review evidence; Broader/Context = interpretation not tested as an imaging-clinical association in that study. NR = not reported in the abstract or bibliographic record examined. ACC, anterior cingulate cortex; ALFF, amplitude of low-frequency fluctuations; CRP, C-reactive protein; DLPFC, dorsolateral prefrontal cortex; DMN, default-mode network; DTI, diffusion-tensor imaging; ECT, electroconvulsive therapy; FC, functional connectivity; HC, healthy controls; HAMD - Hamilton Depression Rating Scale MDD, major depressive disorder; MRS, magnetic resonance spectroscopy; OFC, orbitofrontal cortex; PCC, posterior cingulate cortex; PFC, prefrontal cortex; ReHo, regional homogeneity; SERT, serotonin transporter; sgACC, subgenual ACC; TSPO, translocator protein; VBM, voxel-based morphometry.

### 3.1 Neurotrophic signaling and brain structural and functional changes in MDD

Neurotrophic signaling refers to interconnected molecular pathways that regulate neuronal growth, survival, synaptic remodeling, and adaptive plasticity within the central nervous system, primarily through neurotrophins such as brain-derived neurotrophic factor (BDNF) and its downstream signaling cascades, including TrkB, PI3K/Akt/mTOR, and MAPK/ERK pathways (Chao and Bothwell, 2002; Lee and Kim, 2010; Numakawa and Kajihara, 2023). These mechanisms are essential for maintaining normal emotional and cognitive function, as well as for enabling adaptive responses to stress (Numakawa and Kajihara, 2023; Pittenger and Duman, 2008). Impaired neuroplasticity has therefore been proposed as one of the several key pathophysiological mechanisms contributing to MDD pathophysiology (Duman and Aghajanian, 2012; Pittenger and Duman, 2008).

Structural and functional neuroimaging studies have independently reported abnormalities in brain regions that are highly involved in neuroplastic processes and stress regulation, including the hippocampus, prefrontal cortex, and anterior cingulate cortex (Arnone et al., 2013; Drevets et al., 2008; Otte et al., 2016; Schmaal et al., 2016). However, these neuroimaging findings should not themselves be interpreted as evidence of impaired neurotrophic signaling, because most studies did not directly assess neurotrophic markers. Direct studies integrating molecular measures of neurotrophic signaling with neuroimaging outcomes remain relatively limited, and the extent to which alterations in these pathways contribute to the reported structural and functional abnormalities remains unresolved.

#### 3.1.1 BDNF signaling, genetic variability, and neuroimaging alterations in MDD

The neurotrophin family includes four major members, among which BDNF is the most widely studied in the mammalian brain. Neuroplastic processes are differentially regulated by two forms of BDNF: the precursor form (pro-BDNF) and mature BDNF (mBDNF). These isoforms act through distinct receptors: mBDNF binds to the tropomyosin receptor kinase B (TrkB) receptor, activating signaling cascades that support neuronal survival and synaptic strengthening, whereas pro-BDNF interacts with the p75 neurotrophin receptor (p75NTR), which may trigger apoptosis and synaptic pruning (Chao and Bothwell, 2002; Duman et al., 2021).

Disruption of this regulatory balance has been strongly implicated in the pathophysiology of MDD. Numerous studies have reported reduced *BDNF* expression together with dysregulation of its downstream signaling pathways, particularly in the hippocampus and prefrontal cortex, regions closely associated with mood regulation, stress adaptation, and memory processes (Dwivedi et al., 2003). A systematic review and meta-analysis found that peripheral BDNF concentrations are generally lower during acute depressive episodes and tend to increase following successful antidepressant treatment, supporting the involvement of impaired neurotrophic signaling in MDD (Kishi et al., 2017; Sen et al., 2008). However, peripheral BDNF should not be considered a direct surrogate marker of central BDNF activity, as the relationship between circulating and brain BDNF remains incompletely understood (Molendijk et al., 2014).

Separately, neuroimaging studies have reported structural and functional abnormalities in the hippocampus, prefrontal cortex, and anterior cingulate cortex, regions also implicated in neuroplasticity and stress-related processes (Arnone et al., 2013; Schmaal et al., 2016). Nevertheless, because BDNF signaling and neuroimaging measures are generally assessed in separate studies, these findings should be interpreted as converging evidence rather than direct molecular-to-brain relationships. Similarly, imaging-genetic studies investigating the relationship between BDNF genetic variation and brain morphology have produced inconsistent findings, suggesting that molecular and neuroimaging phenotypes cannot yet be linked in a straightforward manner (Pereira et al., 2018; Zhang H.-F. et al., 2018). Only a limited number of studies have simultaneously assessed peripheral BDNF measures and neuroimaging outcomes. For example, serum BDNF concentrations have been associated with regional cortical thickness and gray matter volume in patients with depression (Polyakova et al., 2020). In addition, peripheral BDNF promoter methylation has been associated with cortical thickness in recurrent MDD, providing direct multimodal evidence of an association between epigenetic variation in BDNF and brain structure (Na et al., 2016). More recently, multimodal approaches integrating serum BDNF with resting-state fMRI have suggested that combining peripheral biomarkers with neuroimaging may improve the characterization of MDD, although these findings do not establish direct mechanistic relationships between circulating BDNF and brain network abnormalities (Chen S. et al., 2023).

Beyond alterations in *BDNF* expression itself, increasing attention has focused on downstream signaling pathways regulated by *BDNF*, particularly the BDNF/TrkB and PI3K/Akt/mTOR cascades, which play key roles in neuronal survival, synaptic plasticity, and cognitive function. However, direct evidence linking alterations in these signaling pathways to neuroimaging abnormalities in MDD remains limited. Importantly, experimental evidence indicates that the effects of BDNF are highly region-specific. Whereas reduced BDNF signaling within the hippocampus and prefrontal cortex is generally associated with impaired synaptic plasticity and depressive-like phenotypes, increased BDNF activity within mesolimbic reward circuits has been shown to promote stress susceptibility and depressive-like behaviors in experimental models, suggesting that the biological consequences of BDNF dysregulation depend on the neural circuits involved (Yu and Chen, 2011).

In addition to altered neurotrophic signaling activity, genetic variability within BDNF-related pathways may contribute to individual differences in MDD susceptibility, treatment response, and neuroimaging phenotypes. Among the most extensively studied variants is the *BDNF* Val66Met polymorphism, which has been associated with altered activity-dependent BDNF secretion, antidepressant response, and stress sensitivity. Neuroimaging studies further suggest that the Val66Met polymorphism may influence amygdala-cortical structural covariance and resting-state functional connectivity during adolescence, with stronger effects observed in females, indicating that the neurobiological effects of BDNF genetic variation may depend on developmental stage and sex (Wheeler et al., 2018). However, recent meta-analytic evidence indicates that the association between the Val66Met polymorphism and MDD susceptibility varies across populations and remains inconsistent across studies, highlighting considerable genetic heterogeneity (Wang et al., 2023). While some reports demonstrate associations between the Val66Met variant and treatment-related changes in *BDNF* expression, others fail to identify a direct link with clinical improvement, highlighting the complex interplay between genetic, environmental, and neurobiological factors in MDD (Hong et al., 2010; Kang et al., 2010). Some structural MRI studies have reported smaller hippocampal volumes in Met allele carriers than in Val homozygotes (Frodl et al., 2007). However, subsequent systematic reviews and meta-analyses have not consistently replicated this association, indicating substantial variability across imaging-genetic studies (Harrisberger et al., 2015; Pereira et al., 2018).

Variants in the *NTRK2* gene have likewise been associated with structural and clinical features of MDD, including reduced hippocampal volume, treatment resistance, and increased risk of suicidal behavior (Deflesselle et al., 2018; Paolini et al., 2023). However, the available evidence does not yet establish whether *NTRK2* genetic variation is consistently associated with specific structural or functional neuroimaging phenotypes in MDD, highlighting the need for further multimodal and longitudinal investigation.

#### 3.1.2 ARC signaling and systems-level neuroplastic dysfunction in MDD

In addition to BDNF itself, increasing attention has focused on downstream molecular mediators of neuroplasticity that may link neurotrophic dysfunction to large-scale neural circuit abnormalities in MDD. One of the most extensively studied of these molecules is activity-regulated cytoskeleton-associated protein (ARC), a key downstream effector of BDNF–TrkB signaling involved in synaptic plasticity and long-term potentiation. Functionally, ARC contributes to the maintenance of synaptic structure and neuronal plasticity through cytoskeletal interactions and regulation of dendritic spine dynamics (Gallo et al., 2018; Lyford et al., 1995). As an immediate early gene regulated through the ERK/MAPK pathway, ARC plays a critical role in synaptic function and neuronal communication (Xing et al., 2016). Reduced *ARC* expression has been reported in transcriptomic and postmortem studies of patients with MDD, supporting its involvement in impaired neuroplasticity and disrupted neuronal connectivity (Gammie, 2021; Yu et al., 2022). However, the magnitude of these alterations varies across studies, potentially reflecting differences in experimental models, brain regions examined, disease stage, and methodological approaches.

Disruption of ARC signaling has also been associated with cognitive dysfunction and altered dopaminergic activity, particularly within mesofrontal pathways. Supporting this relationship, chronic stress, one of the major risk factors for MDD, has been shown to reduce *ARC* expression, especially in the medial prefrontal cortex and hippocampus, primarily in experimental models, suggesting a potential role for stress-related neuroplastic impairment in depression (McEwen et al., 2016; Ons et al., 2010). Environmental stressors such as social isolation have likewise been shown to suppress hippocampal *ARC* expression and induce depressive-like behaviors in animal studies (Pisu et al., 2011).

At the systems level, neuroimaging studies have separately reported structural and functional abnormalities involving the prefrontal cortex, hippocampus, and interconnected limbic networks in MDD (Arnone et al., 2013; Jung et al., 2020; Zhang F. et al., 2018; Zheng et al., 2023). However, these studies did not directly assess ARC expression or signaling. Therefore, the molecular and neuroimaging findings should be interpreted as converging evidence across different levels of neuroplastic dysfunction rather than as demonstrating a direct ARC–neuroimaging relationship. Direct multimodal evidence linking ARC alterations to structural or functional brain abnormalities in patients with MDD remains lacking.

Genetic and transcriptomic evidence further supports the role of ARC-related neuroplastic dysfunction in MDD. Reduced *ARC* gene expression has been consistently identified in transcriptomic studies of depression, while specific single nucleotide polymorphisms (SNPs), including rs11167152 and rs7465272, have been associated with symptom severity and suicidal ideation, respectively (Crisafulli et al., 2021; Gammie, 2021). However, the reported genetic associations remain heterogeneous across studies, which may reflect differences in sample size, ancestry, clinical characteristics, and transcriptomic methodologies.

Beyond ARC-mediated neuroplastic dysfunction, additional neurotrophic signaling pathways involving p75NTR, AKT, and MAPK signaling further support the concept of impaired neuroplasticity as a systems-level feature of MDD. Alterations in the proBDNF/p75NTR system under chronic stress conditions may contribute to synaptic dysfunction, neurodegeneration, immune activation, and neuroinflammatory responses; however, most supporting evidence originates from experimental studies Genetic association studies have further suggested variations in genes such as *AKT1* and dysregulation of PI3K-Akt, MAPK, and JAK–STAT pathways have been associated with symptom severity, antidepressant response, and potential biomarker signatures in MDD (Fujii et al., 2011; Gao et al., 2021; Losenkov et al., 2016; Yang et al., 2024; Yang, 2012). Nevertheless, these findings remain heterogeneous and require replication in larger, well-characterized cohorts. Direct evidence linking alterations in these signaling pathways with specific neuroimaging phenotypes in MDD also remains limited.

### 3.2 Stress-related neuroendocrine dysregulation and brain structural-functional alterations in MDD

#### 3.2.1 HPA axis dysregulation and stress-related network abnormalities

The interaction between chronic stress and neuroplasticity plays a central role in the neuroendocrine mechanisms underlying MDD. Studies in rodents have shown that prolonged stress exposure can induce persistent dysregulation of the hypothalamic–pituitary–adrenal (HPA) axis together with reductions in neurotrophic support, which may contribute to weakened synaptic connectivity and reduced resilience to stress. Both experimental and clinical studies have reported reduced BDNF expression in MDD. Antidepressant treatment has been associated with partial restoration of BDNF levels, suggesting that stress-related neuroplastic alterations may be, at least in part, reversible (Lee and Kim, 2010; Molendijk et al., 2014).

In addition to BDNF, other neuropeptides involved in stress-related neuroplasticity, including VGF, a neuropeptide precursor involved in neuroplasticity and energy homeostasis, and neuropeptide Y have also been implicated in MDD. Experimental studies have shown that reduced expression of these neuropeptides has been associated with impaired executive function and increased stress sensitivity, while antidepressant treatment has been reported to partially restore their expression (Jiang et al., 2018; Mathé et al., 2020). Nevertheless, findings remain heterogeneous, and evidence in humans is comparatively limited.

Activation of the hypothalamic–pituitary–adrenal (HPA) axis is accompanied by increased production of corticotropin-releasing hormone (CRH) and adrenocorticotropic hormone (ACTH), leading to sustained cortisol secretion and persistent activation of stress response pathways (Kageyama et al., 2021; Pariante and Lightman, 2008). HPA axis abnormalities are not uniformly observed across all individuals with MDD, and alterations in cortisol secretion, glucocorticoid receptor function, and negative feedback sensitivity vary across clinical subtypes, early-life stress exposure, treatment status, and methodological approaches, suggesting substantial biological heterogeneity (Ceruso et al., 2020; Juruena et al., 2018; Nandam et al., 2019).

Separately, neuroimaging studies have reported structural and functional abnormalities in stress-sensitive regions and circuits, including reduced hippocampal volume, prefrontal cortical alterations, altered amygdala structure, and disrupted connectivity within limbic circuits involved in stress regulation and emotional processing (Arnone et al., 2013; Belleau et al., 2019; Shin et al., 2025; Zhang F. et al., 2018). However, because these studies did not simultaneously assess HPA-axis markers, these findings should be interpreted as converging systems-level evidence rather than direct evidence that HPA-axis dysregulation underlies the observed brain abnormalities.

Importantly, a small number of multimodal studies have directly examined this relationship by simultaneously assessing peripheral cortisol concentrations and structural neuroimaging measures. These studies have demonstrated associations between serum cortisol levels and cortical thinning (Liu et al., 2015), white matter integrity (Liu et al., 2016), hippocampal shape abnormalities (Watanabe et al., 2017), and structural brain network alterations (Nguyen et al., 2020) in first-episode, drug-naïve patients with MDD. Although these findings provide more direct evidence linking HPA-axis activity with brain structure, the small number of studies and their focus on specific clinical populations limit their generalizability.

Within this neuroendocrine framework, CRH is considered a central mediator of HPA axis activation, and its involvement in MDD has been extensively investigated. While CRH is necessary for adaptation to acute stress, chronic elevation of CRH signaling has been associated with adverse emotional, cognitive, and physiological outcomes. Biochemical and clinical studies support the presence of HPA axis hyperactivity in depression (Claes, 2004). Notably, one longitudinal neuroimaging study found that normalization of HPA axis dysfunction following pharmacotherapy was accompanied by changes in neural activity within the prefrontal cortex and limbic/paralimbic regions, suggesting an association between endocrine regulation and functional brain changes in MDD (Aihara et al., 2007). At the molecular level, *CRH* expression is tightly regulated by glucocorticoid-mediated negative feedback mechanisms involving glucocorticoid receptors and associated regulatory proteins, including cyclic adenosine monophosphate (cAMP) response element modulator and suppressor of cytokine signaling 3 (SOCS3). Disruption of these regulatory pathways, especially involving the glucocorticoid receptor gene (*NR3C1*), has been implicated in impaired feedback regulation and sustained HPA axis activation and increased susceptibility to depression (Kageyama et al., 2021; Shim et al., 2022; Van West et al., 2006). In addition to its neuroendocrine effects, dysregulation of CRH signaling may contribute to systemic comorbidities associated with MDD. Hypercortisolism has been proposed as a shared mechanism linking depression with metabolic disorders such as type 2 diabetes mellitus (T2DM) (Perrelli et al., 2024). Variants in corticotropin-releasing hormone receptor genes, *CRHR1* and *CRHR2*, may further contribute to both metabolic and neuroendocrine dysfunction, while interactions between *CRHR1* and BDNF signaling have been associated with altered stress responsiveness, hippocampal vulnerability, and treatment outcomes in recurrent MDD (Xiao et al., 2011). However, direct multimodal evidence linking CRHR genetic variation to specific structural or functional neuroimaging phenotypes in MDD remains limited.

Functional alterations of the HPA axis are further supported by evidence of abnormal adrenal sensitivity and dysregulated cortisol dynamics. Increased responsiveness to ACTH together with elevated cortisol awakening responses have been reported in both active and remitted MDD, suggesting persistent biological vulnerability even beyond symptomatic episodes (Jaeckle, 1987; Vreeburg et al., 2009). Altered ACTH and cortisol levels have also been associated with symptom severity, such as suicidal ideation, cognitive impairment, and anxiety (Strumila et al., 2024; Tian et al., 2024; Xie et al., 2023). However, the strength of these associations varies across studies, potentially reflecting differences in patient characteristics, treatment status, endocrine assessment protocols, and neuroimaging methodologies.

Collectively, HPA-axis dysregulation represents an important but heterogeneous neuroendocrine feature of MDD. Unlike several other molecular pathways discussed in this review, the HPA axis is supported by a small body of direct multimodal evidence linking cortisol-related abnormalities with structural and functional brain measures. Nevertheless, these studies remain limited in number and population coverage, and the broader correspondence between HPA-axis dysfunction and stress-related brain abnormalities is still largely based on converging evidence from separate endocrine and neuroimaging investigations.

#### 3.2.2 Additional stress-responsive molecular and neuroendocrine mechanisms in MDD

Beyond classical HPA axis dysregulation, additional stress-responsive molecular systems may also be implicated in the pathophysiology of MDD. Immediate early genes (IEGs), including early growth response (EGR) family (EGR1-4), are involved in stress adaptation, synaptic plasticity, and activity-dependent neuronal regulation (Gallo et al., 2018; Honey et al., 2009). Postmortem studies have reported reduced *EGR1* expression in the medial prefrontal cortex of patients with depression has been reported, suggesting a potential role for EGR1 in impaired neuroplasticity and antidepressant response (Covington et al., 2010). In addition, experimental studies have shown that *EGR1* participates in regulating hippocampal activity under stress conditions, and its dysregulation has been linked to altered neural activity and impaired synaptic plasticity (Gallo et al., 2018; Sancho-Balsells et al., 2023). Separately, structural and functional neuroimaging studies have identified abnormalities within cortico-limbic networks involved in emotional processing and cognitive control in MDD (Zheng et al., 2023; Zhu et al., 2023). However, because EGR1 expression and neuroimaging measures have not generally been assessed within the same study populations, these findings should be interpreted as converging evidence rather than evidence of a direct relationship between EGR1 dysregulation and cortico-limbic abnormalities. Direct multimodal evidence linking EGR1 alterations with neuroimaging phenotypes in MDD remains lacking.

Stress-related neuroendocrine dysregulation in MDD may also involve additional endocrine systems, particularly the hypothalamic–pituitary-thyroid (HPT) axis. Alterations in thyroid function, including dysregulated thyroid-stimulating hormone (TSH) levels, have been associated with increased risk of suicidal behavior (Luo et al., 2024). Experimental studies have suggested that altered expression of the thyroid hormone-inactivating enzyme deiodinase 3 (*DIO3*) may impair neurogenesis and synaptic plasticity through dysregulation of thyroid hormone signaling, leading to its proposal as a potential biomarker in MDD (Liang et al., 2025; Pagnin et al., 2021). In contrast to the limited direct evidence for EGR1, structural MRI evidence provides a more direct link between thyroid-related endocrine measures and brain structure: serum TSH levels have been associated with gray matter volume in the middle frontal gyrus, which in turn was associated with executive function in patients with MDD (Zhao et al., 2021). This finding provides multimodal evidence linking an endocrine marker, regional brain structure, and cognitive function; however, evidence integrating HPT-axis molecular markers with neuroimaging outcomes remains limited.

### 3.3 Neuroinflammation and glia-mediated structural-functional brain changes in MDD

Growing evidence suggests that neuroinflammatory processes are involved in the pathophysiology of MDD (Figueroa-Hall et al., 2020; Shariq et al., 2018). In addition to their role in peripheral immune regulation, inflammatory mechanisms within the central nervous system may influence neuroplasticity, synaptic function, neurotransmitter signaling, and large-scale brain network organization. Increasing attention has also focused on glial cells, particularly microglia and astrocytes, which play critical roles in neuroimmune signaling and maintenance of neural homeostasis.

#### 3.3.1 Cytokine dysregulation and large-scale brain network alterations

Cytokines, which regulate immune and inflammatory responses, have emerged as important mediators in the pathophysiology of MDD (Turner et al., 2014).

Increasing evidence suggests that excessive pro-inflammatory signaling may influence neurotransmitter metabolism, synaptic plasticity, glial activity, and large-scale neural network organization, thereby contributing to emotional and cognitive dysfunction in depression (Figueroa-Hall et al., 2020; Jiao et al., 2025; Rahimian et al., 2022). Meta-analyses have generally reported elevated levels of pro-inflammatory cytokines, such as IL-1β, IL-6, IL-18, and TNF-α, in individuals with depression (Köhler et al., 2017). However, inflammatory profiles appear to vary substantially across patient subgroups, suggesting considerable biological heterogeneity within MDD, with elevated inflammatory markers observed only in a subset of individuals with the disorder (Osimo et al., 2020, 2019; Poletti et al., 2024). This variability may reflect differences in disease stage, treatment status, comorbidities, patient characteristics, and methods used to assess inflammatory biomarkers (Pandey et al., 2021). In addition, certain chemokines, such as MCP-2/CCL8, have been associated with specific clinical features, including suicidal behavior, suggesting that inflammatory signaling may contribute to symptom-specific neurobiological alterations in MDD (Liu et al., 2020).

Notably, only a limited number of multimodal studies have simultaneously assessed peripheral inflammatory markers and neuroimaging measures. For example, blood inflammatory markers have been associated with functional network dysconnectivity in patients with depression (Aruldass et al., 2021), while peripheral inflammation has also been linked to alterations in structural and functional connectivity of depression-related brain networks (Kitzbichler et al., 2021). These findings provide direct multimodal evidence of an association between peripheral inflammatory activity and brain network organization; however, they do not establish that peripheral inflammatory abnormalities directly reflect central neuroinflammatory processes or causally drive the observed network alterations. Anti-inflammatory pathways may also play a modulatory role in MDD pathophysiology. Increased expression of *IL10* and other immune-regulatory mediators has been reported in patients with depression, while antidepressant treatment may further enhance IL-10 production (Kubera et al., 2000; Sforzini et al., 2023). However, genetic associations involving *IL10* polymorphisms remain inconsistent across studies, possibly owing to differences in population characteristics, sample sizes, and study design (Koido et al., 2010).

#### 3.3.2 NF-κB and inflammasome signaling in MDD

Inflammatory signaling in MDD is thought to involve activation of Toll-like receptor (TLR)-mediated innate immune pathways. Activation of TLRs triggers intracellular signaling cascades involving the transcription factor nuclear factor kappa B (NF-κB), which regulates expression of multiple pro-inflammatory cytokines and promotes activation of inflammasomes, particularly the NLR family pyrin domain containing 3 (NLRP3) inflammasome (Hung et al., 2016; Kawai and Akira, 2007; Rasmusson et al., 2021; Saleki et al., 2024). Activation of the NLRP3 pathway enhances maturation of inflammatory cytokines such as IL-1β and IL-18 and has been observed in both experimental models and in several clinical studies of MDD, although much of the mechanistic evidence originates from animal and *in vitro* studies, whereas evidence in humans is derived predominantly from peripheral blood biomarkers, with relatively limited direct evidence from postmortem brain tissue or *in vivo* neuroimaging studies (Guo et al., 2015; Kouba et al., 2022; Liu et al., 2017; Taene et al., 2020). However, the reported magnitude of NLRP3 activation varies across studies, potentially reflecting differences in patient populations, disease severity, treatment exposure, and methodological approaches.

Beyond their immunological effects, experimental evidence suggests that NF-κB- and inflammasome-related pathways may also contribute to impaired neuroplasticity and large-scale brain dysfunction in MDD. Separately, neuroimaging studies examining broader processes have reported associations between inflammatory dysregulation and altered activity or functional connectivity in regions including the anterior cingulate cortex and insula, which are involved in affective processing and salience network function in MDD (Aruldass et al., 2021; Han and Ham, 2021). However, these findings should not be interpreted as direct evidence linking NF-κB or NLRP3 signaling to these neuroimaging abnormalities, because these specific pathways were not directly assessed together with neuroimaging outcomes. Direct multimodal evidence linking NF-κB or NLRP3 activity with structural or functional brain alterations in MDD therefore remains lacking. Despite growing evidence implicating NF-κB and NLRP3 signaling in MDD, these pathways have not yet been established as clinically validated biomarkers because of methodological heterogeneity, inconsistent replication across studies, and the limited availability of direct *in vivo* human evidence.

#### 3.3.3 Cytokine-dependent signaling and inflammatory network dysfunction

In addition to NF-κB-mediated inflammatory cascades, cytokine-dependent intracellular signaling pathways have also been implicated in the pathophysiology of MDD. Among these, the Janus kinase/signal transducer and activator of transcription (JAK/STAT) pathway has been implicated in inflammatory signaling relevant to MDD and may influence neuroplasticity, neurogenesis, and antidepressant response (Al-Samhari et al., 2016; Gałecka et al., 2022; Shariq et al., 2018). Experimental studies have shown that stress-induced activation of JAK-3 in the hippocampus is associated with reduced neurogenesis; however, pharmacological inhibition of JAK-3 or antidepressant treatment may partially reverse these effects (Gulbins et al., 2016). Much of the mechanistic evidence is derived from experimental models, whereas evidence in humans remains relatively limited and is based predominantly on peripheral biomarkers rather than direct assessment of brain tissue (Gałecka et al., 2022; Shariq et al., 2018).

Among cytokine-dependent JAK/STAT pathways, the IL-6/IL-6R/STAT3 axis has attracted particular attention in depression research. Studies of peripheral blood samples have reported elevated IL-6, STAT3, and RORγt (retinoic acid receptor-related orphan receptor Gamma t) levels in untreated patients with MDD, whereas antidepressant treatment appears to normalize these alterations (Askari et al., 2019). However, the reported alterations in IL-6/STAT3 signaling vary across studies, potentially reflecting differences in disease severity, treatment status, patient characteristics, and methodological approaches.

At the systems level, neuroimaging studies examining broader inflammatory processes have reported associations between peripheral inflammatory markers and altered structural and functional connectivity in brain networks implicated in MDD (Aruldass et al., 2021; Kitzbichler et al., 2021). However, these studies do not establish that the observed network abnormalities are specifically related to JAK/STAT signaling, as JAK/STAT pathway activity was not directly assessed together with neuroimaging outcomes. Thus, the molecular and neuroimaging findings should be interpreted as converging evidence rather than as demonstrating a direct JAK/STAT–brain relationship. Direct multimodal evidence specifically linking JAK/STAT pathway alterations with neuroimaging phenotypes in MDD remains lacking.

#### 3.3.4 Microglial activation and neuroimmune interactions

Microglia, the resident immune cells of the central nervous system, are thought to play an important role in linking peripheral inflammation to brain dysfunction in MDD. Altered microglial activation has been associated with both inflammatory signaling and impaired neuroplasticity, supporting a potential contribution to the pathophysiology of depression (Rahimian et al., 2022). Postmortem studies have reported region-specific microglial activation, particularly in the anterior cingulate cortex (Torres-Platas et al., 2014). Several PET imaging studies have reported increased translocator protein (TSPO) binding, an indirect marker of glial activation, in the anterior cingulate cortex, prefrontal cortex, and insula during major depressive episodes (Holmes et al., 2018; Setiawan et al., 2015). However, other PET studies have not detected significant differences between patients with MDD and healthy controls (Hannestad et al., 2013), indicating heterogeneity in the *in vivo* imaging evidence for glial-related neuroinflammation in MDD. Postmortem findings have also been inconsistent, with several studies reporting no significant changes in microglial markers or suggesting predominantly homeostatic rather than pro-inflammatory microglial phenotypes (Enache et al., 2019; Snijders et al., 2021).

Chronic stress and sustained inflammatory signaling may shift microglia toward a pro-inflammatory phenotype, potentially contributing to excessive cytokine release, impaired synaptic remodeling, and altered neuron–glia communication (Jiao et al., 2025; Rahimian et al., 2022). Such alterations may disrupt synaptic homeostasis and may be associated with impaired neuroplasticity within stress-sensitive brain regions, particularly the hippocampus and prefrontal cortex (Li et al., 2022; McEwen et al., 2016; Rahimian et al., 2022). Experimental studies further suggest that abnormal microglial activation may influence dendritic remodeling and synaptic pruning, thereby contributing to cognitive dysfunction and emotional dysregulation in MDD (Jiao et al., 2025; McEwen et al., 2016; Rahimian et al., 2022). Nevertheless, much of the mechanistic evidence supporting these observations has been obtained from experimental models, whereas direct evidence in patients with MDD remains comparatively limited.

Importantly, TSPO-PET provides *in vivo* evidence relevant to glial activation, but TSPO binding is not specific to microglia or to a particular pro-inflammatory microglial phenotype. Therefore, the PET findings should be interpreted as evidence of altered glial-related neuroimmune activity rather than as direct evidence of a specific microglial activation state. Together with the inconsistent postmortem findings, this limits conclusions regarding the precise role and phenotype of microglia in MDD.

Notably, inflammatory pathways appear to be closely interconnected with neurotrophic and stress-related mechanisms. For example, NF-κB signaling, in addition to regulating immune responses, also participates in neurogenesis and synaptic plasticity through interactions with BDNF-related pathways (Caviedes et al., 2017). These findings suggest potential interactions between neuroimmune and neurotrophic mechanisms; however, their relationship with specific structural and functional brain abnormalities in MDD remains incompletely established because these pathways are rarely assessed simultaneously with neuroimaging measures.

Overall, accumulating evidence supports a major role for neuroimmune dysregulation in MDD. However, findings remain partially heterogeneous, particularly regarding specific inflammatory profiles and their relationship to clinical subtypes. This variability likely reflects differences in immune responses, disease stages, and methodological approaches across studies. Further multimodal studies combining molecular or cellular markers of neuroimmune activity with neuroimaging measures are needed to determine how glia-mediated mechanisms relate to structural and functional brain alterations in MDD.

### 3.4 Monoaminergic and neurotransmitter systems in brain structural-functional alterations in MDD

Dysregulation of neurotransmitter systems has long been considered a central component of MDD pathophysiology. Neurotransmitters are chemical signaling molecules that mediate synaptic communication and regulate emotional, cognitive, behavioral, and stress-related processes that are frequently disrupted in MDD (Aminoff and Daroff, 2014). Major neurotransmitter systems implicated in MDD include monoamines such as serotonin (5-HT), norepinephrine (NE), and dopamine (DA), as well as excitatory and inhibitory neurotransmitters including glutamate and gamma-aminobutyric acid (GABA).

The monoamine hypothesis represents one of the earliest biological models of depression and proposes that impaired monoaminergic signaling contributes to the development of depressive symptoms (Nutt, 2008). While this framework has played a foundational role in antidepressant development, monoaminergic dysfunction alone does not fully explain the clinical heterogeneity of MDD or the delayed therapeutic response observed with many treatments. Increasing evidence instead suggests that monoaminergic abnormalities interact with broader glutamatergic, GABAergic, and neuropeptide systems, reflecting the complex and multidimensional neurobiology of depression.

The following subsections summarize the roles of major neurotransmitter systems involved in MDD, including monoaminergic (serotonin, norepinephrine, adrenaline, and dopamine), glutamatergic, GABAergic, and neuropeptide-related signaling pathways, together with their associated structural-functional brain alterations.

#### 3.4.1 Serotonin

Serotonin (5-HT) is one of the most extensively studied neurotransmitters in MDD because of its central role in mood regulation, emotional processing, cognition, and stress responsiveness. Altered serotonergic signaling has been implicated in depressive symptom development and has historically formed the basis for many conventional antidepressant strategies (Bhatt et al., 2021). However, increasing evidence suggests that serotonin function extends beyond its classical neurotransmitter role and also influences broader cellular processes, including mitochondrial function, astrocytic regulation, and calcium homeostasis, highlighting its involvement in multiple aspects of neuronal physiology (Cardon et al., 2024). Clinical studies evaluating peripheral serotonin have produced heterogeneous findings. Moreover, peripheral serotonin concentrations do not consistently correspond to measures of central serotonergic activity, indicating that circulating serotonin should not be considered a direct surrogate marker of brain serotonin function (Obermanns et al., 2022). Consistent with these observations, recent umbrella reviews have questioned whether alterations in serotonin alone are sufficient to explain the pathophysiology of MDD, suggesting that serotonergic dysfunction represents one component of a broader and more complex neurobiological framework rather than a universal mechanism (Moncrieff et al., 2023).

More direct investigation of central serotonergic function has been achieved using molecular neuroimaging approaches, particularly PET and SPECT assessment of the serotonin transporter (SERT). PET studies have demonstrated altered interregional SERT availability following selective serotonin reuptake inhibitor treatment in patients with MDD (James et al., 2017). More recently, large-scale multimodal PET-fMRI studies have further characterized the relationship between serotonergic neurotransmission and functional brain organization during emotional processing, highlighting the potential value of molecular neuroimaging for investigating serotonergic dysfunction in depression (Klöbl et al., 2025). However, systematic reviews of PET and SPECT studies have reported substantial heterogeneity in serotonin transporter findings across imaging investigations, limiting their utility as consistent biomarkers of MDD (Kambeitz and Howes, 2015).

Separately, functional MRI studies of SSRI treatment have reported in large-scale functional connectivity, particularly within default mode and fronto-limbic networks, although the reported effects vary considerably across studies (Turiaco et al., 2025). Because these studies assess brain-network changes following serotonergic treatment rather than directly measuring serotonergic signaling together with functional connectivity, they provide complementary treatment-related evidence but should not be interpreted as demonstrating a direct relationship between serotonin availability and network dysfunction.

Genetic variation within the serotonergic system has also been investigated in relation to neuroimaging phenotypes. In particular, the serotonin-transporter-linked polymorphic region (5-HTTLPR) of *SLC6A4* has been examined in imaging-genetic studies of MDD. A systematic review and meta-analysis found no significant association between the 5-HTTLPR short (S) allele and total hippocampal volume, whereas exploratory voxel-wise analyses identified white-matter microstructural abnormalities, predominantly involving the corpus callosum, in MDD patients carrying the S allele compared with non-carriers. However, substantial heterogeneity was observed across studies, indicating that 5-HTTLPR-related neuroimaging findings remain inconsistent (Pereira et al., 2018).

The functional complexity of serotonergic signaling is further reflected by receptor subtype diversity and by mechanisms regulating serotonin availability. For example, the 5-Hydroxytryptamine Type 3 (5-HT3) receptor has been implicated in mood regulation, pain perception, stress responses, and antidepressant effects (Bhatt et al., 2021). Serotonin homeostasis is tightly regulated by both enzymatic degradation and precursor metabolism. Monoamine oxidase (MAO), encoded by *MAOA* and *MAOB* genes, plays a key role in serotonin degradation, while genetic variation in these enzymes may influence antidepressant response (Chappell et al., 2022).

In parallel, tryptophan metabolism through the kynurenine pathway represents another critical regulatory mechanism. Under stress and inflammatory conditions, tryptophan is preferentially metabolized toward kynurenine production, resulting in reduced serotonin synthesis and accumulation of neuroactive metabolites. An imbalance between neurotoxic quinolinic acid and neuroprotective kynurenic acid has been associated with neuroinflammation, impaired neuroplasticity, and depressive pathology (Correia and Vale, 2022; Gong et al., 2023). Genetic factors further contribute to serotonergic dysregulation. Variants in the tryptophan hydroxylase 1 (*TPH1*) gene have been investigated in relation to MDD susceptibility and antidepressant response; however, findings remain heterogeneous across populations and may depend on ethnic, environmental, and clinical factors (Chen et al., 2012; Lian et al., 2013).

Overall, these findings suggest that serotonergic dysfunction in MDD extends beyond a simple monoamine deficiency model and involves complex interactions between neurotransmission, neuroplasticity, inflammation and, metabolism. Although molecular neuroimaging and imaging-genetic studies provide opportunities to examine serotonergic mechanisms in relation to brain structure and function, findings remain heterogeneous, and relatively few studies directly integrate multiple serotonergic measures with neuroimaging and clinical phenotypes within the same individuals.

#### 3.4.2 Epinephrine and norepinephrine

The noradrenergic system plays a key role in emotional regulation, cognitive processes, arousal, attention, and adaptive responses to stress. Norepinephrine (NE), the main neurotransmitter of the locus coeruleus-noradrenergic system and a major mediator of sympathetic activity, modulates neuronal excitability, synaptic transmission, immune responses, and stress-related behavior. Dysregulation of NE signaling has therefore been closely linked to the pathophysiology of MDD, particularly symptoms related to impaired stress adaptation, reduced motivation, emotional instability, and cognitive dysfunction (Moret and Briley, 2011; Zorina et al., 2025). Clinical studies evaluating peripheral norepinephrine and epinephrine concentrations have produced heterogeneous findings. Reported alterations vary across depressive subtypes, illness severity, stress exposure, sampling conditions, and analytical methodologies, indicating that peripheral catecholamine abnormalities are not uniformly observed in all individuals with MDD (Kelly and Cooper, 1998; Maletic et al., 2017; Potter and Manji, 1994).

At the molecular level, noradrenergic signaling is regulated by norepinephrine transporters, adrenergic receptors, and downstream second-messenger pathways. Studies have reported reduced norepinephrine transporter (NET) availability and altered NE binding sites in patients with MDD, suggesting impaired synaptic transmission and disrupted noradrenergic tone (Rady et al., 2022).

Recent experimental evidence further indicates that early-life stress may alter the transcriptional activity of the α2A-adrenergic receptor and its associated cAMP/PKA signaling pathway in the frontal cortex, linking stress exposure to molecular changes in noradrenergic neurotransmission and depression-like behavior (Ali and Dwivedi, 2025). However, these findings are primarily derived from experimental models, and their relevance to human MDD requires further investigation.

Adrenergic receptors represent an important mechanism through which norepinephrine and epinephrine influence emotional and cognitive processes. Variations in adrenergic receptor genes have been implicated in MDD risk, although their functional significance remains incompletely understood (Kokut et al., 2015). In particular, β-adrenergic receptors have been increasingly discussed in relation to depression and stress resilience, as they participate in monoaminergic signaling, neuroplasticity, HPA-axis regulation, and antidepressant response (Zhang et al., 2022). These findings suggest that adrenergic receptor dysfunction may contribute not only to altered mood regulation but also to broader abnormalities in stress-related neurobiological pathways.

Increasing attention has also been given to the α2-adrenergic receptor (α2AR) and its interaction with the adenosine A2A receptor (A2AR). A2AR is expressed not only in the basal ganglia but also in limbic and cortical regions involved in mood regulation and cognitive processing (Fredholm, 2011; Mishina et al., 2007). Experimental studies suggest that α2AR-A2AR interactions may modulate neurotransmitter release, synaptic plasticity, emotional behavior, and stress responsiveness, indicating a potential role in depressive symptomatology (Bevilacqua et al., 2025; Crema et al., 2013; Oliveira et al., 2019). However, most evidence for this receptor-level interaction remains preclinical, and its relevance to human MDD has not yet been established.

Genetic factors may further contribute to variability in noradrenergic function. Polymorphisms in the norepinephrine transporter gene (*SLC6A2*) have been associated with increased susceptibility to MDD and may influence clinical heterogeneity, although findings remain inconsistent across populations. Dopamine-β-hydroxylase (DBH), which catalyzes the conversion of dopamine to norepinephrine, represents another important component of this pathway. Genetic variation in *DBH* may affect enzyme activity and noradrenergic tone, potentially contributing to individual differences in depression risk and treatment response (Hashmi et al., 2024). However, current evidence suggests that noradrenergic-related variants are unlikely to act independently and should instead be interpreted within broader polygenic networks involving stress-response, inflammatory, and neurotransmitter pathways (Wang et al., 2024; Zorina et al., 2025).

Despite substantial molecular, genetic, and pharmacological evidence implicating noradrenergic signaling in MDD, direct multimodal evidence linking specific noradrenergic markers or genetic variants with structural or functional neuroimaging abnormalities remains limited. Consequently, structural and functional brain alterations reported independently in MDD should not be attributed specifically to noradrenergic dysfunction in the absence of concurrent molecular or neurochemical assessment.

The noradrenergic system has also been implicated in treatment response. Modulation of noradrenergic signaling has been associated with the therapeutic effects of electroconvulsive therapy (ECT), while antidepressants that enhance norepinephrine availability may improve depressive symptoms by restoring catecholaminergic transmission (Moret and Briley, 2011; Pollak et al., 2021). Nevertheless, treatment response remains highly heterogeneous, suggesting that noradrenergic dysfunction represents only one component of a broader pathophysiological network.

Collectively, noradrenergic abnormalities represent one component of the heterogeneous neurotransmitter and stress-related alterations reported in MDD. Further multimodal studies combining direct measures of noradrenergic function with neuroimaging and clinical phenotyping are needed to determine whether specific noradrenergic alterations correspond to reproducible structural or functional brain phenotypes in MDD.

#### 3.4.3 Dopamine

Dopamine plays a key role in motivation, reward processing, emotional regulation, and executive function. One of the hallmark symptoms of MDD, anhedonia, has been strongly linked to dysfunction of the dopaminergic signaling pathways and impaired reward-related neural processing.

At the molecular level, dopamine release and signaling are regulated by multiple intracellular and synaptic mechanisms. Synaptotagmin proteins (Syt), especially Syt4 and Syt7, modulate dopamine release and synaptic transmission (Delignat-Lavaud et al., 2022). Notably, overexpression of Syt4 has been shown to suppress BDNF–TrkB signaling and induce anhedonia-like behavior in animal models (Kim et al., 2024), highlighting an important interaction between dopaminergic and neurotrophic mechanisms in depression. Genetic variation affecting dopamine receptors and transporters such as *SLC6A3* has likewise been associated with MDD susceptibility and other neuropsychiatric disorders, suggesting pleiotropic effects across reward-related conditions (Reith et al., 2022). Additional intracellular regulators such as PRKCB and PDE10A further influence dopaminergic tone through modulation of cAMP and cGMP-dependent signaling pathways that regulate frontal-striatal dopaminergic neurotransmission (Heckman et al., 2016). Recent epigenetic studies have additionally suggested that altered *PRKCB* methylation may represent a potential biomarker of first-episode, drug-naïve adolescent MDD, particularly in individuals with childhood chronic stress (Tao et al., 2024). Only a limited number of multimodal studies have simultaneously assessed central dopaminergic function together with neuroimaging measures. Molecular neuroimaging studies combining PET-based measures of dopaminergic function with functional connectivity analyses have reported associations between altered striatal dopaminergic measures and abnormalities in striatal connectivity and reward-related circuitry in MDD (Hamilton et al., 2018; Schneier et al., 2018). These studies provide more direct evidence linking central dopaminergic abnormalities with systems-level brain dysfunction than studies relying on peripheral dopamine measures. However, the available multimodal evidence remains limited, and direct relationships between circulating dopamine levels and structural or functional brain abnormalities remain poorly characterized.

Collectively, these findings suggest that dopaminergic dysfunction may contribute to both molecular and large-scale circuit abnormalities underlying anhedonia, impaired motivation, and cognitive dysfunction in MDD. However, the limited number of direct multimodal studies and differences in the dopaminergic measures and imaging approaches used across studies currently limit conclusions regarding a consistent dopamine-related neuroimaging phenotype in MDD.

#### 3.4.4 GABA (gamma-aminobutyric acid)

According to the GABAergic hypothesis, a core mechanism underlying MDD involves impaired inhibitory neurotransmission and disruption of the balance between excitatory and inhibitory neural activity (E: I balance), primarily mediated by glutamatergic excitatory signaling and GABAergic inhibitory signaling. This balance is essential for maintaining normal emotional, cognitive, and network-level brain function. Reduced GABAergic activity may contribute to mood disturbances, cognitive dysfunction, and treatment resistance, while traditional antidepressants primarily restore inhibitory signaling over time (Della Vecchia et al., 2022; Luscher et al., 2011).

Proton magnetic resonance spectroscopy (^1^H-MRS) provides *in vivo* evidence of regional neurochemical alterations in MDD. MRS studies have generally reported reduced GABA levels in the prefrontal cortex and anterior cingulate cortex (ACC) in patients with current or remitted depression (Godfrey et al., 2018; Ritter et al., 2022). However, findings remain heterogeneous. Individual studies have reported normal prefrontal GABA concentrations in remitted patients, while meta-analyses indicate that the magnitude of GABA abnormalities varies according to illness stage, brain region, and MRS methodology (Godfrey et al., 2018; Hasler et al., 2005; Schür et al., 2016). Alterations in glutamate (Glu) and glutamine (Gln) have also been reported using MRS, suggesting broader abnormalities in excitatory–inhibitory neurochemistry in MDD (Ritter et al., 2022). Nevertheless, MRS measures regional metabolite concentrations rather than synaptic neurotransmission directly, and therefore these findings should not be interpreted as direct measures of functional GABAergic signaling.

Molecular and experimental studies further support the role of GABAergic dysfunction in stress-related neuroplastic abnormalities. In animal models, chronic stress has been shown to decrease GABA levels, reduce the activity of the glutamic acid decarboxylase 67 (GAD67) enzyme, and impair GABAergic interneuron populations, particularly those expressing somatostatin and parvalbumin (Fogaça et al., 2025). Similarly, postmortem transcriptomic analyses have demonstrated reduced expression of GABA-related genes, including *CRH*, mainly in GABAergic neurons of the subgenual anterior cingulate gyrus (sgACC), suggesting region-specific vulnerability of inhibitory circuits in MDD (Oh et al., 2022). Additional molecular regulators such as NPAS4, a transcription factor involved in activity-dependent synaptic plasticity, have also been implicated in impaired inhibitory regulation and altered stress responsiveness in depression (Guidotti et al., 2012; Jaehne et al., 2015; Selçuk et al., 2023). However, these molecular and postmortem findings have generally not been assessed together with *in vivo* MRS or other neuroimaging measures in the same individuals, limiting direct molecular–neuroimaging integration.

Recent therapeutic approaches targeting the GABAergic system have demonstrated promising antidepressant effects. Neuroactive steroids (NASs) that act as positive allosteric modulators (PAMs) of GABA*_A_* receptors, including brexanolone, zuranolone, and PH10, provide further evidence for the therapeutic relevance of GABAergic signaling (Cutler et al., 2023). In addition, ketamine, although primarily known for its glutamatergic effects, also modulates GABAergic interneurons in the medial prefrontal cortex (mPFC) (Fogaça et al., 2025), further supporting the interconnected nature of excitatory, inhibitory, neuroplastic, and stress-related mechanisms in MDD.

Overall, MRS, postmortem, and experimental evidence implicates GABAergic abnormalities in MDD, although their magnitude appears to vary across brain regions, illness stages, and methodological approaches. While MRS provides *in vivo* evidence of regional GABA-related neurochemical abnormalities, direct studies integrating these measures with molecular, genetic, and functional neuroimaging markers remain limited.

#### 3.4.5 Glutamate

Impaired inhibitory control associated with GABAergic dysfunction is often accompanied by alterations in glutamatergic transmission, which plays a central role in the pathophysiology of MDD. As the primary excitatory neurotransmitter in the brain, glutamate is essential for synaptic plasticity, learning, and memory (Pittenger and Duman, 2008; Sanacora et al., 2008).

One of the key mechanisms implicated in depression involves dysregulation of glutamate receptor signaling, particularly through NMDA and AMPA receptors, which regulate synaptic strength and adaptive neuroplasticity. Dysregulation of these pathways has been linked to impaired synaptic connectivity and reduced resilience of neural circuits in MDD. The rapid antidepressant effects of ketamine, an NMDA receptor antagonist, further highlight the critical role of glutamatergic signaling in depressive disorders (Duman and Aghajanian, 2012; Krystal et al., 2013).

*In vivo* proton magnetic resonance spectroscopy (^1^H-MRS) studies have provided evidence of regional glutamatergic abnormalities in MDD, although findings remain heterogeneous. Individual studies have reported no significant differences in cortical glutamate concentrations between medication-free patients with MDD and healthy controls, suggesting that glutamatergic abnormalities may depend on brain region, illness stage, and imaging methodology (Godlewska et al., 2018). A limited number of multimodal studies have directly assessed relationships between peripheral glutamatergic markers and central neuroimaging measures. One study combined serum glutaminase measurements with proton magnetic resonance spectroscopy and reported reduced anterior cingulate glutamate concentrations in medication-free patients with MDD. However, peripheral glutaminase was not significantly associated with central glutamate levels, highlighting the limited availability of reliable peripheral biomarkers of central glutamatergic dysfunction (Wise et al., 2018). This null association is important because it indicates that peripheral glutamatergic measures cannot necessarily be assumed to reflect central glutamatergic abnormalities.

Other multimodal investigations have focused directly on central glutamatergic measures. Combined PET and proton magnetic resonance spectroscopy studies have demonstrated associations between metabotropic glutamate receptor availability and central glutamatergic metabolism, illustrating the value of molecular neuroimaging approaches for investigating glutamatergic dysfunction in MDD (Abdallah et al., 2017). Nevertheless, the number of such studies remains limited, and differences in receptor targets, brain regions, clinical populations, and imaging methodologies constrain the identification of a consistent glutamatergic neuroimaging phenotype.

Genetic studies have identified significant associations between MDD and variants in genes encoding glutamate receptors, including *GRM7*, *GRM8*, *GRIK4*, and *GRIN2B*, suggesting potential contributions to both disease susceptibility and antidepressant response (Li et al., 2016; Sun et al., 2019). Emerging computational approaches have additionally been used to integrate genetic and neuroimaging data and investigate relationships between genetic variation and large-scale brain organization (Lin et al., 2018). However, evidence specifically linking glutamatergic genetic variation to reproducible neuroimaging phenotypes in MDD remains limited. Collectively, MRS, molecular imaging, genetic, and experimental evidence implicates glutamatergic abnormalities in MDD, while substantial heterogeneity remains across brain regions and methodological approaches. Importantly, glutamatergic signaling interacts extensively with GABAergic, monoaminergic, neuroplastic, and stress-related mechanisms, supporting a broader systems-level interpretation of neurotransmitter dysfunction in MDD.

### 3.5 Oxidative stress and mitochondrial dysfunction in structural-functional brain alterations in MDD

Oxidative stress has been implicated in the pathophysiology of MDD, reflecting an imbalance between the production of reactive oxygen species (ROS) and antioxidant defense mechanisms. Experimental, clinical, and review evidence suggests associations between oxidative damage, reduced antioxidant capacity, neuroinflammation, impaired neuroplasticity, and mitochondrial dysfunction in depression (Maes et al., 2011; Ng et al., 2008; Tobe, 2013).

A limited number of multimodal studies have directly examined peripheral oxidative stress-related markers together with neuroimaging measures in MDD. For example, Liu et al. (2024) reported that plasma antioxidant enzyme levels were associated with altered spontaneous brain activity and thalamic functional connectivity in first-episode, drug-naïve patients with MDD. These findings provide direct multimodal evidence of an association between peripheral antioxidant status and functional brain alterations; however, their cross-sectional nature does not establish a causal molecular-to-network relationship, and peripheral antioxidant measures may not directly reflect oxidative processes within the central nervous system (Liu et al., 2024).

One of the central cellular defense mechanisms against oxidative stress is the Keap1-Nrf2-ARE (antioxidant response element) pathway, which regulates antioxidant enzyme expression and maintains redox homeostasis Beyond its antioxidant role, this pathway has been shown to modulate inflammation, cellular survival processes, and mitochondrial integrity, thereby contributing to overall brain homeostasis (Hashimoto, 2018; Yao et al., 2016).

Preclinical studies using chronic stress models have demonstrated reduced expression of Kelch-like ECH-associated protein 1 (Keap1) and nuclear factor erythroid 2-related factor 2 (Nrf2) in brain regions including the hippocampus and prefrontal cortex. Nrf2 knockout models further exhibit elevated pro-inflammatory cytokines levels together with decreased *BDNF* gene expression, linking oxidative stress to both inflammation and impaired neurotrophic signaling. On the contrary, pharmacological activation of Nrf2, using compounds such as sulforaphane (SFN) or its natural precursor glucoraphanin, has been shown to alleviate depressive-like behaviors in experimental models, potentially via modulation of the BDNF–TrkB signaling pathway (Hashimoto, 2018; Yao et al., 2016). However, these findings are predominantly preclinical, and direct multimodal studies specifically assessing Keap1-Nrf2-ARE pathway activity together with neuroimaging outcomes in patients with MDD remain lacking.

Clinical and translational studies additionally indicate that oxidative stress-related molecules may serve as potential biomarkers in MDD. However, findings vary substantially according to the biological specimen, oxidative or antioxidant marker assessed, disease stage, symptom severity, treatment exposure, age, lifestyle factors, and comorbid medical conditions (Ait Tayeb et al., 2023). Consistent with this heterogeneity, large cohort studies have not uniformly demonstrated increased oxidative stress across all biomarkers or patient groups, indicating that oxidative stress abnormalities may characterize only a subset of individuals with MDD (Black et al., 2017). Similarly, a study in children and adolescents with depressive disorder found that most oxidative stress and antioxidant markers did not differ significantly from healthy controls, apart from selected antioxidant enzymes, further illustrating age- and marker-specific variability (Katrenčíková et al., 2021). Experimental studies also suggest biological variability in therapeutic responsiveness. For example, McCallum et al. (2024) reported that activation of Nrf2 with dimethyl fumarate (DMF) reduced microglial activation and produced antidepressant-like effects in male rats, but not in females, suggesting sex-dependent variability in therapeutic responsiveness.

Overall, the Keap1-Nrf2-ARE pathway represents one of several molecular systems implicated in the interactions among oxidative stress, inflammation, and neuroplasticity in MDD. Although initial multimodal evidence links peripheral antioxidant measures with functional brain alterations, direct evidence linking Keap1-Nrf2-ARE pathway activity itself with structural or functional neuroimaging phenotypes in MDD remains lacking.

## 4 Limitations

Several limitations should be considered when interpreting the evidence synthesized in this review. First, there remains insufficient evidence directly linking molecular or peripheral biomarkers of MDD with structural or functional neuroimaging measures. Despite reported molecular, genetic, neuroendocrine, neuroimmune, neurotransmitter, electrophysiological, and neuroimaging abnormalities in MDD, most of these findings have been obtained from separate study populations. Consequently, correspondence between abnormalities observed at different biological levels should generally be interpreted as converging or associative evidence rather than as demonstrating direct molecular-to-brain causal relationships. Moreover, substantial heterogeneity exists across the MDD literature with respect to clinical subtype, illness stage and severity, treatment exposure, comorbidities, age, sex, biological specimens, molecular assays, and neuroimaging methodologies. Furthermore, peripheral biomarkers do not necessarily reflect corresponding processes within the central nervous system. These factors may partly account for inconsistent findings across neurotrophic, neuroendocrine, inflammatory, neurotransmitter, and oxidative-stress pathways and suggest that individual biological abnormalities are unlikely to represent universal mechanisms across all individuals with MDD. Larger longitudinal multimodal studies across diverse and well-characterized populations are therefore needed to determine whether associations between molecular, genetic, and neuroimaging abnormalities are reproducible, how they vary across clinical subgroups, and whether any reflect causal biological relationships.

The strengths of this review include its integrative approach, bringing together evidence from molecular, genetic, neuroendocrine, neuroimmune, electrophysiological, and neuroimaging studies. However, as an integrative narrative review, the study selection process may be subject to selection bias, and relevant evidence may not have been captured comprehensively. In addition, only English-language publications were considered, which may have resulted in the exclusion of relevant studies published in other languages.

## 5 Conclusion

Major depressive disorder is increasingly recognized as involving interconnected dysfunction across large-scale brain networks and molecular pathways, rather than as a condition driven solely by neurotransmitter imbalance. Advances in structural and functional neuroimaging, electrophysiology, genetics, and molecular neuroscience have revealed converging alterations in prefrontal-limbic circuits, neuroplasticity, neurotrophic and stress-related signaling, immune dysregulation, and neurotransmitter systems. Collectively, many of these alterations appear to be dynamic and potentially reversible, highlighting neuroplasticity as an important mechanism associated with therapeutic response.

Despite these advances, the current body of evidence has several important limitations. Findings across studies are often inconsistent due to methodological variability, limited longitudinal data, and heterogeneous patient populations. Moreover, the lack of standardized and clinically validated biomarkers continues to limit diagnostic precision and the implementation of precision psychiatry. The complex interplay between genetic, epigenetic, environmental, and developmental factors further complicates the translation of research findings into clinical practice.

Future research should prioritize large-scale, multimodal, and longitudinal approaches integrating neuroimaging with molecular, genetic, and epigenetic profiling. The application of network-based models and machine learning approaches may improve patient stratification, diagnostic accuracy, and prediction of treatment response. In parallel, therapeutic strategies targeting neuroplasticity, such as pharmacological interventions, psychotherapy, lifestyle-based approaches, and non-invasive neuromodulation techniques, may provide more effective and sustained clinical outcomes. Ultimately, continued integration of neuroimaging, molecular biology, and genomics may support a more biologically informed understanding of MDD and facilitate progress toward precision psychiatry and personalized care.
