# Correction: Neurobiological mechanisms and brain network dysfunction in major depressive disorder: an integrative review

**DOI:** 10.3389/fnint.2026.2027229

**Published:** 2026-10-26

**Authors:** Dina Iskakova, Burcu Yener, Altyngul Kamzanova, Aygul Sitakanova, Rifat Hamoudi, Almira Kustubayeva

**Affiliations:** 1 Department of Biophysics, Biomedicine, and Neuroscience, Al-Farabi Kazakh National University, Almaty, Kazakhstan; 2 Research Institute of Medical and Health Sciences, University of Sharjah, Sharjah, United Arab Emirates; 3 Department of Psychiatry and Narcology, S.D. Asfendiyarov Kazakh National Medical University, Almaty, Kazakhstan; 4 Department of Clinical Science, College of Medicine, University of Sharjah, Sharjah, United Arab Emirates; 5 Biomedically Informed Artificial Intelligence Laboratory (BIMAI-Lab), University of Sharjah, Sharjah, United Arab Emirates; 6 Division of Surgery and Interventional Science, University College London, London, United Kingdom; 7 Brain Institute, Al-Farabi Kazakh National University, Almaty, Kazakhstan

**Keywords:** functional connectivity, genetics, MDD (major depressive disorder), molecular mechanism, neuroimaging

In the published article, Almira Kustubayeva was erroneously not listed as the first corresponding author. The correct order of the corresponding authors is Almira Kustubayeva, followed by Rifat Hamoudi.

In the published article, Almira Kustubayeva and Rifat Hamoudi were erroneously omitted as equal senior contributing authors. The equal senior authorship symbol (‡) and the following statement should have been included.

“‡These authors have contributed equally to this work and share senior authorship”

The original version of this article has been updated.

