# Supplementary material for: Neurobiological mechanisms and brain network dysfunction in major depressive disorder: an integrative review
Source: Front Integr Neurosci. 2026 Sep 29;20:1904444. doi: 10.3389/fnint.2026.1904444 (PMC13659288; doi:10.3389/fnint.2026.1904444)
Supplement: Supplementary file 1 [file Table_1.docx]

| **Study** | **Sample / Clinical Characteristics** | **Imaging Modality** | **Brain Region / Network** | **Main Imaging Finding** | **Clinical Association Reported** |
| --- | --- | --- | --- | --- | --- |
| **A. Primary clinical, treatment, familial-risk and multimodal cohort studies** | | | | | |
| **Abdallah et al., 2017** | 30 medication-free MDD; 35 HC (PET). Subset: 16 MDD; 23 HC (¹H-MRS). | [¹⁸F]FPEB PET + ¹H-MRS | ACC; whole-brain mGluR5 | No group difference in mGluR5; MDD had higher ACC glutamate, glutamine and Glx. mGluR5 availability inversely related to glutamate/Glx. | Direct—PET–MRS association tested in the same subset; no symptom-severity association reported. |
| **Agziyart et al., 2024** | 60 MDD; 60 HC. Episode and medication status NR. | Resting-state fMRI; graph theory | SMN, DAN, DMN, visual and ventral-attention networks | Lower global/local efficiency and nodal degree/efficiency; longer path length in MDD. | Group only—no study-specific symptom or outcome association reported. |
| **Aihara et al., 2007** | 24 antidepressant-free MDD; 10 treatment responders rescanned; HC comparison (n NR in abstract). | [¹⁸F]FDG-PET + DEX/CRH test | Medial PFC; hippocampal/parahippocampal regions | Frontal hypometabolism and right hippocampal/parahippocampal hypermetabolism; abnormalities normalized in responders. | Direct—metabolism related to HPA-axis status and treatment response in this cohort. |
| **Arnone et al., 2013** | 39 medication-free current unipolar depression; 25 medication-free remitted; 66 HC. Thirty-two current cases received citalopram for 8 weeks. | T1 MRI; VBM | Bilateral hippocampus | Lower hippocampal gray matter in current depression; volume increased after treatment/remission. | Direct—state and longitudinal treatment/remission association. |
| **Aruldass et al., 2021** | 83 depression (CRP ≤3 mg/L, n=50; >3 mg/L, n=33); 46 HC. | Resting-state fMRI + blood inflammatory markers | Interoceptive network (insula-centered) | High-CRP depression showed attenuated functional connectivity. | Direct—connectivity associated with CRP/immune markers in this sample. |
| **Ayyash et al., 2021** | CAN-BIND-1 source cohort: 164 MDD; 103 HC; analyzed fusion subset NR in abstract. | Structural MRI + resting-state fMRI fusion | DMN and ventral-attention network | Small case–control differences in anatomically weighted functional connectivity. | Group only—no participant-level clinical association reported. |
| **Cakmak et al., 2022** | 12 treatment-resistant MDD (concomitant medication); 23 HC. | Hybrid [¹⁸F]FEPPA PET/MRI + DTI | Left sgACC; insula; cingulum | Higher sgACC TSPO uptake, lower sgACC–insula functional connectivity, and structural-connectivity abnormalities. | Direct—sgACC TSPO uptake predicted HAMD severity; preliminary small-sample result. |
| **Chai et al., 2015** | 36 unaffected children at familial MDD risk; 14 low-risk controls; age 8–14 years. | Emotional-face task fMRI + structural MRI | Amygdala; ACC; supramarginal cortex | Risk group showed altered emotional-face activation and smaller amygdala volume. | Direct familial-risk group effect—not a clinical MDD cohort and not a symptom association. |
| **Chen et al., 2023** | 63 MDD; 81 HC. Blood subset: 37 MDD; 45 HC. | Resting-state fMRI + serum proteins | Whole-brain ALFF/ReHo abnormalities | Combined imaging and serum features produced high diagnostic discrimination in-sample. | Direct diagnostic model—association tested in this cohort; not evidence of causality or external clinical validity. |
| **Fan et al., 2025** | 84 MDD/HC participants in discovery cohort before/after ECT (group split NR in abstract); independent 35-MDD validation cohort. | Resting-state fMRI; CVBOLD and FC | Left angular gyrus/precuneus; right lingual cortex | ECT increased left angular/precuneus BOLD variability; precuneus–lingual FC increased. | Direct—FC increase correlated with HAMD improvement; replicated directionally in an independent MDD cohort. |
| **Frodl et al., 2007** | 60 MDD; 60 HC; medication/episode details NR in abstract. | Structural MRI + BDNF Val66Met genotyping | Hippocampus; amygdala | Smaller hippocampi in MDD and in Met-allele carriers; no amygdala effect. | Direct genotype–volume association; no study-specific symptom association reported. |
| **Ge et al., 2021** | 15 severe MDD receiving magnetic seizure therapy; 10 HC. | Longitudinal structural + resting-state fMRI | sgACC–parietal; anterior hippocampus–PFC | Treatment changed functional connectivity and parietal gray matter. | Direct—sgACC–parietal FC increase tracked improvement; hippocampal–PFC decrease tracked poorer clinical/cognitive improvement. |
| **Godlewska et al., 2018** | 55 medication-free depressed participants; 50 HC. | 7-T ¹H-MRS | ACC; occipital cortex; putamen | No glutamate difference; approximately 12% higher putamen glutamine in depression. | Group only—no study-specific symptom or outcome association reported. |
| **Grieve et al., 2013** | 102 non-geriatric MDD outpatients; 34 matched HC; 31% melancholic; mean illness duration 11.3 years. | T1 MRI; whole-brain VBM and cortical thickness | PFC/ACC; precuneus; temporal, parietal and occipital cortex; medial OFC | Widespread lower gray-matter volume and focal left medial OFC thinning. | Negative—structural alterations were not significantly associated with depressive symptom severity. |
| **Hamilton et al., 2018** | 16 depressed participants; 14 HC. | Concurrent [¹¹C]raclopride PET + resting-state fMRI | Ventral/dorsal striatum; DMN and salience targets | Higher striatal binding potential in depression; binding covaried with corticostriatal connectivity. | Direct PET–fMRI association; no symptom-severity association reported. |
| **Hannestad et al., 2013** | 10 acute mild–moderate MDD; 10 genotype-matched HC. | [¹¹C]PBR28 TSPO PET | Whole brain; predefined cortical/subcortical ROIs | No evidence of elevated TSPO; most MDD participants showed lower binding than matched controls. | Negative—no support for increased neuroinflammation; no clinical correlation reported. |
| **Hasler et al., 2005** | 16 fully remitted MDD; 15 HC. | ¹H-MRS | Prefrontal cortex | Prefrontal GABA was not reduced in remitted MDD. | Negative/state-specific—no direct symptom association; result concerns remitted, not acutely depressed, participants. |
| **Holmes et al., 2018** | 14 medication-free moderate/severe MDD; 13 matched HC; post-hoc suicidal-thought subgroup comparison. | [¹¹C](R)-PK11195 TSPO PET | ACC; PFC; insula | Higher TSPO in MDD, significant in ACC; greatest elevations in patients with suicidal thoughts. | Direct—ACC/insula TSPO was associated with suicidal thinking in this cohort; subgroup analysis was post hoc. |
| **James et al., 2017** | 19 MDD outpatients; 19 HC; baseline, acute SSRI challenge, and ≥3-week SSRI follow-up. | [¹¹C]DASB PET | ACC/insula and amygdala, midbrain, hippocampus, pallidum, putamen | No baseline group difference; prolonged SSRI treatment reorganized interregional SERT covariance. | Direct treatment effect—no study-specific symptom-response association reported. |
| **Jung et al., 2020** | 61 MDD; 59 HC; MDD stratified by suicide-attempt history. | Resting-state fMRI; ICA/FNC | Insula, DMN, cerebellum, basal ganglia, thalamus and frontoparietal networks | Widespread network-synchronization deficits; additional insula–DMN/cerebellar dysconnectivity with attempt history. | Direct—network measures related to suicidal ideation/stress; not explained by medication or illness duration. |
| **Katsuki et al., 2020** | 18 initially drug-naïve MDD responders with continuous remission ≥6 months; no HC group. | Longitudinal T1 MRI; hippocampal subfields | Left CA3 and granule-cell/dentate-gyrus subfields | Volumes increased after 6 months of antidepressant treatment. | Direct longitudinal remission/treatment association; no untreated control group. |
| **Kitzbichler et al., 2021** | 83 depression (CRP >3 mg/L, n=33; <3 mg/L, n=50); 46 HC. | Proton-density MRI + DTI + resting-state fMRI | Precuneus/PCC, mPFC, hippocampus; DMN | CRP-related microstructural and connectivity changes colocalized with reduced DMN hubness. | Direct—inflammation–imaging associations tested in the same sample. |
| **Klöbl et al., 2025** | 153 participants, including 44 MDD; randomized acute IV citalopram/placebo. | Emotional-face fMRI + [¹¹C]DASB PET | ACC; striatum/thalamus; parietal/superior frontal cortex | Citalopram reduced fear-related ACC activation; MDD showed altered frontoparietal activation. | Direct—ACC activation inversely related to SERT availability and drug-related activation changes related to emotional attribution. |
| **Kochunov et al., 2025** | Four cohorts: UK Biobank 2,220 recurrent MDD/2,590 controls; ENIGMA 2,148/7,957; ACP 68 lifetime MDD/136 controls; ACI 296 recurrent MDD/76 controls. | Resting-state fMRI ReHo; cortical thickness; external perfusion validation | Cingulate, superior temporal and frontal cortex | Replicable lower ReHo without significant cortical-thickness differences; spatially aligned with lower cerebral blood flow. | Direct—ReHo-derived vulnerability index associated with higher symptom severity across cohorts. |
| **Kustubayeva et al., 2020** | 60 MDD; 60 HC. | Task EEG/ERP; alpha asymmetry | Frontal, central and parietal scalp regions | MDD showed altered lateralization during decision making under positive/negative feedback. | Group/task association—clinical severity or treatment relevance was not established. |
| **Kustubayeva et al., 2023** | 30 melancholic MDD; 21 HC. | Implicit emotional-face fMRI | Thalamus; superior/middle frontal; supramarginal, fusiform, temporal and cingulate regions | Predominantly reduced activation in melancholic MDD, with sex-dependent cingulate/precuneus effects. | Direct—IDS severity inversely related to subgenual/rectal-gyrus activation in women; other claims are group effects. |
| **Liu X. et al., 2015** | 30 drug-naïve first-episode MDD; 41 matched HC. | Structural MRI; cortical thickness + serum cortisol | Left lateral OFC | MDD showed OFC thinning and higher morning cortisol. | Direct—OFC thickness inversely correlated with cortisol in MDD. |
| **Liu X. et al., 2016** | Drug-naïve MDD and matched HC (same first-episode research program; exact analyzed n NR in abstract). | DTI; tract-based spatial statistics + serum cortisol | Distributed white-matter tracts | Lower white-matter integrity was linked to HPA-axis abnormality. | Direct—white-matter measures associated with serum cortisol in the patient sample; not a symptom association. |
| **Liu P. et al., 2021** | 74 first-episode drug-naïve MDD (50 with somatic symptoms; 24 without); 70 HC. | Resting-state fMRI; ReHo/ALFF | Bilateral pre/postcentral and left paracentral cortex | Somatic-symptom MDD showed lower local activity than nonsomatic MDD. | Direct—regional abnormalities negatively related to somatic and depressive symptom severity. |
| **Liu Y. et al., 2024** | 57 first-episode drug-naïve MDD; 60 HC; MDD stratified by suicidal ideation. | Resting-state fMRI + plasma oxidative-stress markers | Thalamus, ACC, superior frontal gyrus, insula and precuneus | SOD/GSR related to regional activity and thalamic connectivity; patterns differed by suicidal-ideation status. | Direct biomarker–imaging association; negative for HAMD association. |
| **Lu et al., 2016** | 30 first-episode untreated MDD; 26 HC. | Structural MRI; volume and shape analysis | Bilateral putamen; left thalamus | Lower volumes and focal surface contraction in MDD. | Direct—left dorsal-thalamic atrophy was negatively associated with clinical severity. |
| **Na et al., 2016** | 65 recurrent MDD; 65 matched HC. | Structural MRI + BDNF promoter methylation | Frontal, temporal, occipital, parietal and sensorimotor cortex | Higher BDNF methylation related to lower cortical thickness across multiple regions in MDD. | Direct epigenetic–imaging association; no treatment-outcome association. |
| **Nguyen et al., 2020** | 42 drug-naïve first-episode MDD; 39 HC. | T1 MRI; source-based morphometry + serum cortisol | Prefrontal and insula–temporal structural networks | Both networks differed in MDD; cortisol was higher. | Direct—cortisol inversely correlated with prefrontal-network loading in MDD. |
| **Paolini et al., 2023** | 121 MDD inpatients; treatment-response/resistance cohort; no HC. | 3-T structural MRI + NTRK2 genotyping | Hippocampus | An NTRK2 variant was associated with hippocampal volume and antidepressant response. | Direct—hippocampal volume statistically mediated the genotype–response relationship; observational association. |
| **Polyakova et al., 2020** | 20 minor depressive episode; 40 HC; first and recurrent episodes represented. | Structural MRI + serum BDNF | OFC, rostral ACC, insula, cingulate and superior frontal cortex | BDNF positively related to regional thickness/volume in depression, driven mainly by first episode. | Direct—but the clinical group had minor depressive episodes, not necessarily DSM MDD. |
| **Ritter et al., 2022** | 251 HC; 98 past MDD; 47 current MDD; predominantly medication-free. | 3-T ¹H-MRS | Left DLPFC | Lower GABA in current and past MDD; past MDD also showed lower glutamate and higher glutamine. | Direct state/history comparison; no participant-level treatment association. |
| **Roddy et al., 2021** | 80 MDD; 83 HC; most MDD participants medicated. | T1 MRI; amygdala-subnuclei segmentation | Medial amygdala; corticoamygdaloid transition area | Larger right medial nuclei/asymmetry; left transition-area volume inversely related to cortisol-awakening response. | Direct HPA-axis–volume association; not a symptom-severity association. |
| **Saylam et al., 2006** | 24 MDD drug-free for ≥4 weeks; 24 HC. | Structural MRI volumetry | Hippocampus; amygdala | Smaller hippocampal volumes, especially left, in MDD. | Direct—right hippocampal volume correlated with HAMD severity. |
| **Schindler et al., 2019** | 20 unmedicated MDD; 20 medicated MDD; 21 bipolar disorder; 23 HC. | 7-T structural MRI | Hypothalamic subunits | Approximately 5% larger left hypothalamus across patient groups. | Direct diagnosis/medication comparison—effect was independent of medication; no symptom association reported. |
| **Schneier et al., 2018** | 26 treatment-naïve MDD; 26 HC; MDD received 6 weeks of pramipexole. | Reward fMRI + [¹¹C](+)PHNO PET; amphetamine challenge | Striatum and reward circuitry | Dopamine-release/receptor metrics were not significantly related to reward BOLD abnormalities. | Negative—no association with severity, anhedonia, or treatment response. |
| **Setiawan et al., 2015** | 20 medication-free MDD in a major depressive episode; 20 HC. | [¹⁸F]FEPPA TSPO PET | ACC; PFC; insula | Higher TSPO distribution volume in MDD. | Direct—ACC TSPO was positively associated with depression severity. |
| **Sheline et al., 2010** | 18 unmedicated MDD; 17 HC; HAMD ≥18 for MDD. | Resting-state fMRI | DMN, cognitive-control and affective networks; dorsomedial PFC ‘dorsal nexus’ | All three networks showed increased connectivity to the dorsal nexus in MDD. | Group only—the proposed link to rumination and other symptoms is a broader mechanistic interpretation, not a tested symptom correlation in this study. |
| **Shin et al., 2025** | 39 depression; 73 HC; medication/episode details NR in abstract. | 3-T structural MRI; hypothalamic subunits | Bilateral inferior tubular hypothalamus; left hypothalamus | Larger hypothalamic subunits in depression; non-linear change with illness duration. | Direct—volume associated with illness-duration/chronicity pattern, not symptom severity. |
| **Tang et al., 2024** | 183 MDD; 300 HC. | Multimodal structural/functional MRI + DNA methylation | Cortical and subcortical SC–FC coupling | Lower global and cortical but higher subcortical structure–function coupling in MDD. | Direct—HPA-axis-related CpG methylation associated with coupling in the same cohort. |
| **Wagner et al., 2011** | 15 high-suicide-risk MDD; 15 low-risk MDD; 30 HC. | T1 MRI; VBM | Fronto-striato-limbic regions; caudate; rostral ACC | High-risk MDD showed lower gray-matter density than low-risk MDD/HC. | Direct—association was with predefined suicide-risk status; not a contemporaneous symptom-severity correlation. |
| **Watanabe et al., 2017** | First-episode drug-naïve MDD and HC; exact analyzed n NR in abstract. | Structural MRI; hippocampal shape + serum cortisol | Hippocampus | Focal hippocampal-shape abnormalities accompanied elevated cortisol. | Direct—hippocampal shape related to serum cortisol; not a treatment-outcome association. |
| **Wheeler et al., 2018** | 339 youth aged 8–22; population/risk cohort, not an MDD case–control sample. | Structural covariance + resting-state fMRI + BDNF genotype | Amygdala–cortical networks | Adolescent Met carriers, especially females, showed stronger amygdala–cortical covariance/connectivity. | Direct risk association—linked to greater likelihood of MDD in adolescent females; not an imaging finding from a clinical MDD cohort. |
| **Wise et al., 2018** | 29 medication-free depression (20 unipolar, 9 bipolar); 20 HC. | ¹H-MRS + peripheral glutaminase assay | Dorsal ACC | Lower ACC glutamate, significant in unipolar depression. | Negative—peripheral glutaminase was not significantly related to central glutamate; clinical sample mixed unipolar/bipolar. |
| **Xue et al., 2024** | 1,112 total: 212 MDD, 380 schizophrenia, 330 bipolar disorder, 190 HC. | Resting-state EEG microstates | Whole-scalp microstate networks | MDD shared altered A↔B and A↔C transition probabilities with other diagnoses. | Group only—transdiagnostic classification result; no MDD-specific symptom association reported. |
| **Yang et al., 2015** | 27 untreated first-episode MDD; 27 HC. | Structural MRI; cortical thickness | Prefrontal and parietal cortex | Greater regional cortical thickness in untreated first-episode MDD. | Negative—thickness did not correlate with anhedonia. |
| **Yi et al., 2025** | 246 MDD (123 with suicidal ideation; 123 without); 235 HC. | Structural MRI/VBM; REST-meta-MDD | Bilateral ACC | Suicidal-ideation MDD showed greater ACC gray-matter volume than non-ideation MDD and HC. | Direct—ACC volume associated with suicidal ideation in this cohort. |
| **Yuan et al., 2024** | 48 MDD; 38 HC; EEG at baseline and after 1 week of antidepressant treatment. | Emotional-face EEG; functional-connectivity analysis | Distributed emotion-processing network | MDD showed lower connectivity at baseline and week 1; no significant early normalization. | Group/early-treatment result—no confirmed symptom-response association reported in the abstract. |
| **Zhang C. et al., 2024** | 42 MDD; 42 HC. | Resting-state fMRI global FC + normative transcriptomic mapping | PCC/precuneus; cerebellar Crus I/II | Lower PCC/precuneus and higher cerebellar global connectivity in MDD. | Direct—left cerebellar connectivity inversely associated with perceived social support; gene links are broader/normative. |
| **Zhao et al., 2021** | 104 MDD; 75 HC. | Structural MRI + thyroid indices + executive testing | Left gyrus rectus; bilateral middle frontal gyrus | Reduced frontal gray matter in MDD. | Direct—TSH associated with gray matter/executive performance; right middle frontal volume mediated the TSH–executive association. |
| **Zheng et al., 2023** | 66 first-episode drug-naïve adolescents with MDD; 47 HC. | Resting-state fMRI; degree centrality | Bilateral hippocampus; left superior temporal gyrus; right insula | Lower regional degree centrality in MDD. | Direct—hippocampal degree centrality related to HAMD severity and illness duration. |
| **Zhu et al., 2023** | Two independent MDD imaging datasets; exact n NR in abstract; includes first-episode drug-naïve subgroup. | Resting-state fMRI ALFF + normative transcriptomics | Distributed cortical/subcortical regions | First-episode drug-naïve MDD showed more extensive ALFF abnormalities. | Group/subtype effect—gene-expression mapping used normative tissue and is not a patient-level molecular association. |
| **Zorina et al., 2025** | Participant n and clinical composition NR in abstract/bibliographic record. | Task EEG + candidate-gene analysis | Inhibitory-control/stop-signal network | Reported gene-related electrophysiological differences during inhibitory control. | Unclear clinical applicability—sample details and an MDD-specific clinical association cannot be verified from the available record. |
| **B. Meta-analyses, systematic reviews and imaging-focused narrative reviews** | | | | | |
| **Belleau et al., 2019** | Narrative review of stress/MDD morphology; no single cohort. | Structural MRI | Hippocampus; medial PFC | Reviews stress- and illness-related variation in hippocampal and medial-prefrontal morphology. | Broader interpretation—no study-specific participant association. |
| **Drevets et al., 2008** | Narrative neuroimaging review; no single cohort. | Structural MRI, fMRI, PET | MPFC/ACC, limbic–striatal–pallidal–thalamic circuits | Synthesizes structural, metabolic and functional abnormalities into mood-circuit models. | Broader interpretation—no study-specific participant association. |
| **Enache et al., 2019** | Systematic review/meta-analysis: 69 CSF, PET and post-mortem studies. | TSPO PET (plus non-imaging evidence) | ACC; temporal cortex | Pooled PET evidence indicated higher TSPO in ACC and temporal cortex in MDD. | Pooled—not an individual-level clinical association; suicidal-behavior result came mainly from CSF evidence. |
| **Fitzgerald et al., 2008** | Activation meta-analysis of resting, treatment and emotion-processing depression studies; study/participant totals NR in abstract. | PET/SPECT/fMRI activation studies | Frontal, temporal, insular, limbic, subcortical and cerebellar regions | Convergent network-level hypo-/hyperactivation and treatment-related normalization. | Pooled/broader—no participant-level association. |
| **Godfrey et al., 2018** | Systematic review/meta-analysis; study/participant totals NR in abstract. | ¹H-MRS | ACC and other spectroscopy voxels | Lower GABA overall and lower ACC GABA/Glx; no significant glutamate difference. | Pooled—not a study-specific severity or outcome association. |
| **Hamilton et al., 2012** | Activation meta-analysis of negative-information processing in MDD; totals NR in abstract. | Task fMRI/PET | Pulvinar, amygdala, insula, dACC, dorsal striatum and DLPFC | MDD showed limbic/salience hyperactivity and dorsal regulatory/reward hypoactivity. | Pooled/broader—no individual-level clinical association. |
| **Han & Ham, 2021** | Narrative review; no single cohort. | Structural/functional MRI and inflammation measures | Corticolimbic networks | Reviews links between peripheral inflammation and MDD-related brain alterations. | Broader interpretation—no study-specific association. |
| **Harrisberger et al., 2015** | Meta-analysis of 18 psychiatric cohorts; 1,695 patients; not MDD-specific. | Structural MRI + BDNF Val66Met | Hippocampus | No significant Val66Met–hippocampal-volume association across clinical cohorts. | Pooled negative result—not an MDD-specific participant association. |
| **Kaiser et al., 2015** | Meta-analysis: 27 datasets/25 publications; 556 MDD; 518 HC. | Resting-state fMRI connectivity | DMN, frontoparietal-control and affective networks | Pooled DMN hyperconnectivity and imbalanced control-network connectivity. | Pooled—clinical interpretations are network-level, not participant-level correlations. |
| **Kambeitz & Howes, 2015** | Meta-analysis: 50 imaging/post-mortem studies; 877 depression; 968 HC. | PET/SPECT SERT imaging (plus post-mortem) | Striatum, amygdala, brainstem, thalamus and hippocampus | Lower SERT availability in striatum, amygdala and brainstem; no thalamic/hippocampal effect. | Pooled—not a study-specific clinical association. |
| **Kishi et al., 2017** | Review of meta-analyses; no single imaging cohort. | Structural MRI/genetics (among broader BDNF evidence) | Hippocampus | Val66Met was not associated with hippocampal volume in MDD in pooled evidence. | Pooled negative imaging-genetic result; broader treatment conclusions came from non-imaging evidence. |
| **Meyer, 2012** | Narrative molecular-neuroimaging review; no single cohort. | PET receptor/transporter imaging | Serotonergic and monoaminergic targets | Summarizes molecular targets, occupancy and implications for treatment development. | Broader interpretation—no study-specific patient association. |
| **Olbrich & Arns, 2013** | Comprehensive EEG review; no single cohort. | Resting/task EEG and ERP | Frontal alpha and distributed electrophysiological markers | Reviews diagnostic discrimination and prediction of antidepressant response. | Broader/predictive literature—not one prospectively validated cohort. |
| **Pereira et al., 2018** | Systematic review: 65 studies (4,034 MDD; 3,293 HC); meta-analytic subset 22 studies (882 MDD; 616 HC). | Imaging genetics; structural/functional MRI | Hippocampus and distributed gray/white-matter and activation phenotypes | No robust BDNF/5-HTTLPR hippocampal-volume effect; exploratory effects elsewhere. | Pooled—no participant-level clinical association; many findings exploratory. |
| **Scheepens et al., 2020** | Systematic review: 14 multimodal MDD studies. | Structural + functional MRI | DMN and connected structural pathways | Most consistent pattern was lower structural integrity with increased DMN connectivity. | Pooled/broader—not an individual-level clinical association. |
| **Schmaal et al., 2016** | Individual-participant meta-analysis: 1,728 MDD; 7,199 HC across 15 cohorts. | Structural MRI; subcortical segmentation | Hippocampus and other subcortical regions | Smaller hippocampus in MDD, driven by recurrent illness; no first-episode effect. | Pooled moderators—early onset/recurrent status related to volume; no severity association. |
| **Schür et al., 2016** | Meta-analysis: 40 studies; 1,591 participants across disorders; MDD n=437. | ¹H-MRS | Cortical GABA/glutamate voxels | Lower GABA in currently depressed MDD but not remitted MDD. | Pooled state association—not an individual-level symptom correlation. |
| **Turiaco et al., 2025** | Systematic review: 11 articles; treatment-effect samples 198 MDD/219 HC; prediction samples 159 MDD, 97 HC, 22 placebo, 56 nonresponders. | Resting-state fMRI | DMN, salience and frontolimbic connectivity | Escitalopram-related FC normalization; baseline FC showed potential for response prediction. | Pooled evidence—predictive relevance is across heterogeneous studies, not one validated biomarker. |
| **Wang et al., 2024** | Meta/transcriptomic analysis: 34 MDD imaging studies (1,621 MDD; 1,507 HC); schizophrenia also analyzed. | Cortical-thickness MRI meta-analysis + transcriptomics | Cortical regions with thickness alteration | Identified genes spatially associated with cortical-thickness abnormalities across disorders. | Pooled spatial association—not patient-level gene expression or a clinical outcome association. |
| **Zhang F. et al., 2018a** | Narrative psychoradiology review; no single cohort. | Structural MRI, fMRI, DTI, PET/MRS | Distributed mood and cognitive networks | Summarizes imaging markers proposed for diagnosis and treatment prediction. | Broader interpretation—no study-specific association or validated clinical test. |
| **Zhang H.-F. et al., 2018b** | Systematic review: 64 neuroimaging-genetics studies; 28 variants. | Structural/functional MRI + genetics | Hippocampal, corticolimbic and distributed networks | Genetic effects were heterogeneous and generally not independently replicated. | Pooled/broader—not a participant-level clinical association. |

**Evidence labels:** Direct = the association was tested in the same participant sample; Negative = the stated clinical association was tested but not supported; Group only = an imaging difference was reported without a study-specific clinical correlation; Pooled = meta-analytic/systematic-review evidence; Broader/Context = interpretation not tested as an imaging–clinical association in that study. NR = not reported in the abstract or bibliographic record examined.

**Abbreviations:** ACC, anterior cingulate cortex; ALFF, amplitude of low-frequency fluctuations; CRP, C-reactive protein; DLPFC, dorsolateral prefrontal cortex; DMN, default-mode network; DTI, diffusion-tensor imaging; ECT, electroconvulsive therapy; FC, functional connectivity; HC, healthy controls; HAMD — Hamilton Depression Rating Scale MDD, major depressive disorder; MRS, magnetic resonance spectroscopy; OFC, orbitofrontal cortex; PCC, posterior cingulate cortex; PFC, prefrontal cortex; ReHo, regional homogeneity; SERT, serotonin transporter; sgACC, subgenual ACC; TSPO, translocator protein; VBM, voxel-based morphometry.
